# Generalisations of a Bayesian decision-theoretic randomisation procedure and the impact of delayed responses

**DOI:** 10.1016/j.csda.2021.107407

**Published:** 2022-10

**Authors:** S. Faye Williamson, Peter Jacko, Thomas Jaki

**Affiliations:** a Department of Mathematics and Statistics, Lancaster University, UK; b Biostatistics Research Group, Population Health Sciences Institute, Newcastle University, UK; c Department of Management Science, Lancaster University, UK; d Berry Consultants, UK; e MRC Biostatistics Unit, Cambridge University, UK

**Keywords:** Bayesian decision-theoretic model, Clinical trials, Delayed responses, Dynamic programming, Response-adaptive randomisation

## Abstract

The design of sequential experiments and, in particular, randomised controlled trials involves a trade-off between operational characteristics such as statistical power, estimation bias and patient benefit. The family of randomisation procedures referred to as Constrained Randomised Dynamic Programming (CRDP), which is set in the Bayesian decision-theoretic framework, can be used to balance these competing objectives. A generalisation and novel interpretation of CRDP is proposed to highlight its inherent flexibility to adapt to a variety of practicalities and align with individual trial objectives. CRDP, as with most response-adaptive randomisation procedures, hinges on the limiting assumption of patient responses being available before allocation of the next patient. This forms one of the greatest barriers to their implementation in practice which, despite being an important research question, has not received a thorough treatment. Therefore, motivated by the existing gap between the theory of response-adaptive randomisation (which is abundant with proposed methods in the immediate response setting) and clinical practice (in which responses are typically delayed), the performance of CRDP in the presence of fixed and random delays is evaluated. Simulation results show that CRDP continues to offer patient benefit gains over alternative procedures and is relatively robust to delayed responses. To compensate for a fixed delay, a method which adjusts the time horizon used in the optimisation objective is proposed and its performance illustrated.

## 1 Introduction

In this paper, we build on the randomisation procedure introduced in Williamson et al. (2017), namely, Constrained Randomised Dynamic Programming (CRDP), which was motivated by the need to develop a randomisation procedure that would improve patient benefit *within* a clinical trial while retaining satisfactory statistical operating characteristics. This is often the case in rare disease settings, for example, where a substantial proportion of all patients with the disease may be included in the trial. Williamson et al. (2017) developed a novel model, set in the Bayesian decision-theoretic framework, in which they introduced a constraint (to ensure a minimum sampling requirement is satisfied) and randomisation into an otherwise deterministic procedure based upon a dynamic programming (DP) solution to obtain an adaptive patient randomisation procedure ahead of the trial implementation.

The family of CRDP procedures forms a continuum of randomisation procedures for the design of randomised controlled trials (RCTs) or, more generally, sequential experiments. Specifically, the CRDP continuum ranges from the traditional fixed randomised procedure from the frequentist framework (which, in theory, provides an unbiased maximum likelihood estimator of the treatment effect) to the response-adaptive procedure from the Bayesian decision-theoretic framework computed by DP (which, in theory, provides the maximal Bayes-expected patient benefit). Williamson et al. (2017) illustrated that CRDP allows for efficiently balancing the competing operational characteristics of statistical power (owing to the use of constraining), estimation bias of the maximum likelihood estimator (owing to the use of randomisation), and patient benefit (owing to the use of DP).

In Section 2, we introduce the methodological framework and generalise CRDP, enhancing its flexibility to adapt to a variety of practicalities. In particular, using our description of the CRDP procedure, the trialist is able to tailor the randomisation procedure by specifying: (1) the time-horizon (the number of future patient allocations that should be taken into account when deciding upon the randomisation probabilities for the current patient), (2) the degree of randomisation (by setting a minimum randomisation probability on each arm, and to do so subject-by-subject or per stages), and (3) the degree of constraining (by specifying trial situations that should be penalised and thus likely avoided). We also provide an alternative representation of CRDP to aid interpretation in subsection 2.3.

Motivated by the existing gap between the theory of response-adaptive randomisation (RAR) (which is abundant with clinical trial design proposals in the setting of immediate responses) and clinical practice (in which responses are typically delayed), we consider CRDP in the presence of delayed responses in Section 3 and Section 4. This is paramount since “if a scheme is impracticable then, no matter what its theoretical advantages happen to be, it will not be used” (Upton and Lee, 1981). In this paper, we take a pragmatic approach with the objective of addressing this problem by not only exploring the impact of delayed responses, but also presenting an adjustment to CRDP to deal with delayed responses.

We will refer to the previously allocated patients, whose responses are not available before allocation of the next patient, as being in the *pipeline*, in keeping with the terminology used in related literature (e.g. Hampson and Jennison, 2013; Chick et al., 2017; Alban et al., 2018). One simple approach is to base the randomisation procedure only on the currently observed data and ignore the pipeline data, which can often lead to biased parameter estimates and incorrect allocation decisions (Xu and Yin, 2014). However, in Section 3, we illustrate that CRDP continues to maintain a good balance between the competing operational characteristics even in this case. Although other attempts to balance patient benefit and power have previously been published (e.g. Mozgunov and Jaki, 2020), as for the majority of RAR procedures, most of these approaches do not account for a delay in response.

We explore the impact of fixed and random delays (i.e. when there is a fixed and random number of patients in the pipeline) on CRDP and compare its performance to the benchmark procedures outlined in subsection 2.4. A fixed number of pipeline patients at each stage will be imposed when the time between two consecutive allocations (i.e. the time period) is constant and patients are followed up at a fixed time after treatment (e.g. Facey, 1992; Whitehead, 1993). If recruitment time or response time (or both) is random, a random number of pipeline patients will arise instead.

There seems to be a common opinion that “adaptive allocation has no benefit when there are long delays” (Berry and Stangl, 1996, Chapter 4) because there is little, or no, chance to adapt the allocation, thus it would be inappropriate to employ a response-adaptive procedure. Our results partly contradict that. We observe that this is true in the extreme case when the delay is nearly equal or greater than the trial size, but only when the delay is assumed to be fixed. When the delay is random, our results illustrate that significant patient benefit is obtained even if the expected delay is greater than the trial size. Roughly speaking, the performance of the CRDP procedure when the delay is very small is the same regardless of whether the delay is fixed or random; but for larger delays, randomness in delays brings performance benefits.

Similarly to Chick et al. (2017, 2020), we then focus on the case where there is a fixed number of patients in the pipeline and suggest an intuitive approach, based on adjusting the time horizon used in the optimisation problem, to account for this delay in Section 4. Finally, the main conclusions and limitations are discussed in Section 5.

To summarise, our contributions are as follows:

- We develop a generalisation of CRDP motivated by clinical trial practicalities;
- We provide an alternative interpretation of CRDP as a bi-level randomisation procedure between a fixed randomised branch and a response-adaptive (deterministic) branch;
- We evaluate CRDP in the presence of fixed and random delayed responses;
- We suggest an adjustment to CRDP in the fixed delay setting to reflect the effect of delay in the optimisation model.

In the rest of this section, we briefly review the literature on RAR and outline some of the few approaches that have considered delayed responses.

### 1.1 Response-Adaptive Randomisation (RAR)

In contrast with the traditional approach adopted in randomised clinical trials, in which the (possibly unequal) treatment allocation probabilities remain constant throughout the trial, *response-adaptive randomisation* (RAR) can be used, in which the randomisation probabilities change during the trial as patient responses are observed. The ability to use this information dynamically as it accrues in order to improve efficiency and allocate more patients to the better performing treatments, for example, makes RAR a particularly attractive mode of patient allocation (Pallmann et al., 2018; Burnett et al., 2020). As stated in the adaptive designs guideline by the U.S. Food and Drug Administration (2018), patients may be more willing to enrol in trials that use RAR, thereby increasing speed and ease of recruitment which poses one of the most challenging aspects in the conduct of traditional RCTs (e.g. Sully et al., 2013). For recent reviews of RAR procedures in clinical trials, refer to Williamson (2020, Chapter 2) and Robertson et al. (2020). Response-adaptive procedures have also been developed in other scientific disciplines, where they are typically referred to as policies or algorithms for the *multi-armed bandit problem;* for a review, see Jacko (2019b).

The RAR procedure discussed in this paper is set in the Bayesian framework in which sequential updating of the unknown parameters, and consequently the allocation probabilities, takes place throughout the trial via repeated application of Bayes' Theorem. Two classes of RAR procedures developed in the Bayesian framework are: (1) those based on the posterior probability of an arm performing better than another, which originate from Thompson (1933) and are commonly referred to as *Bayesian adaptive randomisation*, and (2) those developed using *Bayesian decision theory*, which originate from Bellman (1956). In this paper, the focus is on the latter.

RAR procedures which build on Bayesian decision theory aim to ascertain the optimal treatment allocation based on some desirable criterion, i.e. maximising the expected total reward in the trial. This reward is commonly taken to be a measure of patient benefit, as in CRDP, but not always; for example, Chick et al. (2020) consider cost-benefit improvements of health interventions (i.e. value-based criteria) instead. Bayesian decision-theoretic procedures include policies or solutions to Bayesian bandit problems which are typically response-adaptive, but deterministic; see Villar et al. (2015a) for a review of their utility in the clinical trial context. When these procedures are modified to be non-deterministic (randomised), as in Cheng and Berry (2007) or Williamson et al. (2017), the resulting randomised variants are often referred to as *bandit-based* RAR procedures. These designs have recently re-emerged in the Biostatistics literature with an emphasis on making them more practicable. An important feature of these procedures is that they are non-myopic (or forward-looking), so not only do they adapt to past observations, but also to known future information such as the number of patients remaining to be treated (inside or outside the trial); this is key to optimising the stated objective. Computationally, these procedures can be obtained using DP, the Gittins index or the Whittle index (Villar et al., 2015a; Villar, 2018; Jacko, 2019b). A variety of bandit-based RAR procedures have been evaluated or proposed to address several practicalities, including group-sequential randomisation (Villar et al., 2015b), constrained randomisation (Williamson et al., 2017), covariates (Villar and Rosenberger, 2018), time trends (Villar et al., 2018), continuous outcomes (Smith and Villar, 2018; Williamson and Villar, 2020) and multiple correlated arms in dose-finding trials (Chick et al., 2020).

### 1.2 Delayed responses

To take full advantage of the benefits offered by RAR, each patient's response should ideally be available before the next patient enters the trial to allow for sequential updating of the randomisation probabilities. As such, most designs which incorporate RAR are typically formulated assuming so-called “immediate” patient responses (Cheung et al., 2006; Biswas et al., 2008, Section 3.7). Although this may be appropriate for some clinical contexts, such as trials of surgical interventions (Rosenberger and Lachin, 2016, Chapter 12), emergency medicine trials (Flight et al., 2017), trials for diseases with a slow recruitment rate (e.g. rare diseases) or rapidly observed endpoint (e.g. acute diseases), it is unrealistic in many clinical trial settings (e.g. oncology trials). This is because, not only may a treatment take a substantial length of time to induce a response (e.g. survival trials), but there may also be an administrative delay in obtaining the response (Pocock, 1983) or implementing the adaptation to the allocation probabilities which, as Wason et al. (2019) discusses, “will reduce the efficiency advantage of an adaptive approach in exactly the same way as using an outcome that takes longer to observe”. However, as Biswas and Coad (2005) commented, “most of the available literature on adaptive designs overlooks possible delays in responses”. The inability of most response-adaptive designs to account for delay has long been cited as one of the greatest limitations and barriers to their implementation in practice (e.g. Simon, 1977; Armitage, 1985; Shrestha and Jain, 2021). Sverdlov et al. (2012) describe it as “a major stumbling block in implementing adaptive designs”, and Rosenberger et al. (2012, Section 4) list it as one of the main criticisms of RAR. As such, there is a strong interest amongst the statistical and clinical trial community in how RAR methods perform in the presence of delayed responses and how they can be extended to accommodate for delays.

Several authors have illustrated the *effect* of delayed responses on response-adaptive designs (predominantly urn models), either by simulation, e.g. Robinson (1983); Rosenberger (1999); Ivanova and Rosenberger (2000); Zhang and Rosenberger (2007); Wason et al. (2019), or theoretically, e.g. Bai et al. (2002) for urn models; Hu et al. (2008) for the doubly adaptive biased coin design. These studies showed that although the skewness of the allocation proportion decreases as the delay length increases, these procedures still result in more patients randomised to the better treatment(s).

However, few papers have provided potential solutions to accommodate for the delay, which forms a long-standing open problem. Successful research attempts have focused on simplified problems. For example, Eick (1988a,b); Wang (2000, 2002) studied delayed responses in the context of a two-armed clinical trial where the distribution of one arm is assumed known; Hardwick et al. (2006) presented a simplified solution which reduces the computational requirements, and showed that a procedure based on DP performs very similarly whether responses are delayed or immediate; Xu and Yin (2014) proposed a two-stage non-parametric fractional scheme based on RAR to address the issue of delayed response by treating unobserved responses as censored and calculating their fractional contribution to the response probability; Chick et al. (2020, Section 7.1) proposed a simple heuristic to account for short delays based on batch allocations for a value-based trial. Further examples can be found in Biswas and Bhattacharya (2016, Section 6).

Moreover, the assumption of a fixed-duration delay has been used in almost all of the methodological papers discussing response-adaptive procedures under delayed responses (e.g. Langenberg and Srinivasan, 1981, 1982; Chick et al., 2017, 2020). Fixed delays are mostly a theoretical imperative in order to provide mathematically and computationally tractable approaches. In practice, this can be translated as deterministically regular patient recruitment and deterministically fixed observation times, that is, a constant time period and fixed time until response. This may occur due to recruitment challenges or administrative delays, such as staff availability, resource limitations, time taken to obtain the results, time taken to update and implement the adaptations, etc.

Many clinical trials fit this profile. We provide a few examples below from different disease areas. Tamura et al. (1994) implemented the delayed randomised play-the-winner rule (see Section 2.4) in a two-arm trial for depressive disorder, where the primary outcome was change in the Hamilton Depression Scale from baseline after approximately eight weeks of treatment. They used an intermediate binary surrogate outcome, which was observed sooner than the primary outcome, to adapt the treatment allocation ratios instead. In a randomised two-arm Phase III trial for breast cancer, the primary outcome was occurrence of a pathological complete response (yes/no) six weeks after completion of neoadjuvant therapy (Hurvitz et al., 2018). In stroke trials, the modified Rankin Scale (mRS) at 90 days post-randomisation (which is typically dichotomised to form a binary endpoint) is commonly used as the primary outcome (Ovbiagele et al., 2010). For example, the Pragmatic Ischaemic Stroke Thrombectomy Evaluation (PISTE) trial (Muir et al., 2017) was a RCT comparing intravenousthrombolysis (IVT) alone with IVT plus mechanical thrombectomy in patients with acute ischaemic stroke. The primary outcome was the proportion of patients with favourable functional outcome (defined by a mRS score of 0-2 at day 90).

## 2 Methods

In this section, we introduce the methodological framework and present a generalised version of the CRDP procedure (Williamson et al., 2017), which is constructed based on a formal optimality criterion using the Bayesian decision-theoretic approach cast as a Markov decision process (MDP). With this approach, prior information on the unknown treatment parameters is used in conjunction with the incoming data and, importantly, with the number of remaining patients in the trial to determine the better randomisation probability vector, out of two pre-specified randomisation vectors, for each patient. Note that despite having only two randomisation vector options, the theory of MDPs assures that this is sufficient since adding another randomisation vector in between the original two would not improve the optimal objective, and such an option would be optimal only if both the original two randomisation vectors were optimal.

### 2.1 Bayesian framework for the design of RCTs

We consider a two-armed RCT with a binary endpoint and a finite number of patients within the trial, *n.* Although we use two arms for simplicity of exposition, the framework can be generalised to *K* arms. Patients enter the trial sequentially (one-by-one) over time, and are allocated to either treatment *A* or *B* upon arrival. We assume that *n* is fixed but that the sample sizes for treatment groups *A* and *B*, denoted by *N_A_* and *N_B_* respectively, are random, where *N_A_* + *N_B_* = *n*. We use *t* to denote both time and the last patient treated in this model since they are analogous, that is, at time *t* we have treated *t* patients. The trial time is therefore bounded by 0 ≤ *t* ≤ *n*. Note that in this section, we assume that the response of patient *t* is available before the arrival of patient *t* + 1.

Let *X*_*A*,*t*_ and *X*_*B*,*t*_ denote the patient’s response (either a success or failure) from treatments *A* and *B* respectively, which we model as independent Bernoulli random variables *X*_*j*,*t*_ ∼ Bernoulli(*θ_j_*), where 0 ≤ *θ_j_* ≤ 1 is the unknown success probability of treatment *j* ∈ {*A*, *B*}. We assign the following independent Beta prior distributions to the unknown success probabilities of treatment *j*

$$\theta_{j}∼\text{Beta}\left(s_{j,0},f_{j,0}\right)\;\text{for}\;0\leq \theta_{j}\leq 1,$$

where *s*_*j*,0_ (*f*_*j*,0_) represents the prior number of successes (failures) on treatment *j* at time *t* = 0. Through conjugacy, the resulting posterior distribution thus follows another Beta distribution with parameters summarising the relevant information from the trial to date. That is, at time *t* ≥ 1, after observing *s*_*j*,*t*_ (*f*_*j*,*t*_) successes (failures) on treatment *j*,

$$\theta_{j}|s_{j,t},f_{j,t}∼\text{Beta}\left(s_{j,0}+s_{j,t},f_{j,0}+f_{j,t}\right),$$

where $\sum_{j}s_{j,t}+f_{j,t}=t.$ Let the prior information and data combined be denoted as

$${\tilde{s}}_{j,t}=s_{j,0}+s_{j,t},{\tilde{f}}_{j,t}=f_{j,0}+f_{j,t}.$$

Let *δ*_*j*,*t*_, for *t* = 0, …, *n* − 1, be the binary indicator variable representing whether patient *t* + 1 is allocated to treatment *j*, where

$$\delta_{j,t}=\{\begin{array}{cc} 1, & \text{if}\;\text{patient}\;t+1\;\text{is}\;\text{allocated}\;\text{to}\;\text{treatment}\;j,\\ 0, & \text{otherwise}. \end{array}$$

### 2.2 Generalisation of CRDP

#### 2.2.1 Specification of parameters

In this subsection, we propose a generalisation of the CRDP procedure which allows for specifying the following sets of parameters according to the overall goal and practicalities of the trial.

The time *horizon T* is defined as the end of the time interval which includes all events utilised in the optimisation of the randomisation procedure, i.e. the allocations, observations and any others the trialist may wish to include. Williamson et al. (2017) consider a time horizon equal to the trial size, *T* = *n*, which corresponds to including the benefit of the trial patients and any constraining penalties (described below) that occur during the trial, or just after the observation of the final trial patient. However, the horizon is not restricted to this and could take a range of other values. For example, *T* = 0 means the procedure is completely ignorant of the present and future, *T* = 1 gives rise to a *myopic* design which only considers the present (i.e. the allocation and observation of the next patient). Practicalities may lead to taking *T* smaller than n, e.g. with the aim of optimising the trial patient benefit only over the next stage of the trial, or because of possible patient dropouts or delayed observations which effectively reduce the number of times the randomisation procedure needs to be updated. This is explored in Section 4. A horizon larger than *n* may also be relevant if one wishes to incorporate what happens *after* the trial, in which case *T* will include patients both inside *and* outside the trial, so that the optimal criterion is defined for the entire patient population (or part of it) instead. Since the size of *T* influences how the procedure attains a compromise between the power and patient benefit trade-off, and contributes to the optimality criterion, it is therefore an important element in the specification of such procedures (see e.g. Upton and Lee, 1976; Zhang et al., 2019).

The *degree of randomisation* is defined by a set of parameters 0 ≤ *p*_*j*,*t*_ ≤ 1 for each arm *j* and each time 0 ≤ *t* < *n*, with *p*_*j*,*t*_ = 1 corresponding to a certain (deterministic) allocation to arm *j*. Thus, when *p*_*j*,*t*_ = 1 for all arms *j* and all patients *t*, the DP procedure (which maximises the Bayes-expected patient benefit) is recovered. In the two-arm setting, the actions are defined such that, under action 1, patient *t* + 1 is allocated to treatment *A* with probability *p*_*A*,*t*_ and to treatment *B* with probability 1 − *p*_*A*,*t*_; under action 2, patient *t* + 1 is allocated to treatment *B* with probability *p*_*B*,*t*_ and to treatment *A* with probability 1 − *p*_*B*,*t*_ (or equally randomised between these two actions if they both lead to the same objective value). In practice, we would generally take *p*_*j*,*t*_ ≥ 0.5, although this is not a theoretical requirement. Note that *p*_*j*,*t*_ = 0.5 for both arms *j* in a two-arm trial and for all patients *t* corresponds to fixed equal randomisation. Williamson et al. (2017) only considered the case in which *p*_*j*,*t*_ = *p* for every arm and patient and, from their computational experiments, suggested setting *p* = 0.9.

The *degree of constraining* is defined by a set of trial states which are undesirable and thus should be avoided by the randomisation procedure, together with their corresponding set of penalties. For example, Williamson et al. (2017) considered a penalty of *n* (equivalent to a reward of −*n*) in all the end-of-trial states resulting in fewer than *ℓ* observations per arm, i.e. *s*_*A*,*t*_ + *f*_*A*,*t*_ < *ℓ* or *s*_*B*,*t*_ + *f*_*B*,*t*_ < *ℓ*, with the aim of avoiding extreme allocation imbalance and, consequently, improving statistical power and mitigating estimation bias when the degree of randomisation *p* approaches 1. Several other penalised trial states, or constraints, could be included to restrict the combination of states to those which satisfy some desirable constraint. For example, a penalty could be added to end-of-trial states in which the power is below a particular threshold, or the statistical significance of evidence (*p*-value) is very close to the significance level used in hypothesis testing to avoid borderline decisions.

#### 2.2.2 Formulation

The formulation of CRDP as a Markov decision process can be found in Williamson et al. (2017) but, for completeness, the corresponding generalised objective function is shown below. The value-to-go function *ℱ*_*t*_(***z***) represents the maximum Bayes-expected total reward (i.e. the maximum Bayes-expected number of successes minus constraining penalties) in the remaining time horizon after *t* ≥ 0 patients have been treated when the joint data is ***z*** = (*s*_*A*_, *f*_*A*_, *s*_*B*_, *f*_*B*_) and is given by

(1) $$\begin{array}{l} {ℱ}_{t}\left(\text{z}\right):=\underset{\pi \in \Pi }{\max{𝔼}^{\pi }}\left[Q\left({\text{z}}_{t}\right)+\sum_{u=t+1}^{T}\left(\sum_{j\in \left\{A,B\right\}}\delta_{j,u}X_{j,u}+Q\left({\text{z}}_{u}\right)\right)|{\text{z}}_{t}=\text{z}\right],\\ \;=\underset{\pi \in \Pi }{\max{𝔼}^{\pi }}\left[\sum_{u=t}^{T-1}\left(R^{a_{u}}\left({\text{z}}_{u}\right)+Q\left({\text{z}}_{u}\right)\right)+Q\left({\text{z}}_{T}\right)|{\text{z}}_{t}=\text{z}\right], \end{array}$$

where Π is the family of admissible randomisation procedures *π*, which are such that $\sum_{j}\delta_{j,t}=1$ for all 1 ≤ *t* ≤ *n* (i.e. every patient is allocated to exactly one arm). If we are not at the end of the horizon, i.e. 0 ≤ *t* < *T*, the expected (one-period) reward in state ***z*** under action *a* is given by *R*^a^(***z***). Recall that we receive a reward of 1 for every successfully treated patient, which is given by a product of the random variables *δ*_*j*,*t*_*X*_*j*,*t*_ for patient 1 ≤ *t* ≤ *T*. To calculate its expectation one period ahead, i.e. in period 0 ≤ *t* < *T* for patient *t* + 1, we use the *p*_*j*,*t*_’s as probabilities for *δ*_*j*,*t*+1_ and the Bayesian expectation (current belief) $\frac{{\tilde{s}}_{j,t}}{{\tilde{s}}_{j,t}+f_{j,t}}$ for *X*_*j*,*t*+1_. That is,

$$R^{a_{t}}\left({\text{z}}_{t}\right)=𝔼\left[\sum_{j\in \left\{A,B\right\}}\delta_{j,t+1}X_{j,t+1}|{\text{z}}_{t},a_{t}\right],$$

which can be expressed, for action 1, as

$$R^{1}\left({\text{z}}_{t}\right)=p_{A,t}\cdot \frac{{\tilde{s}}_{A,t}}{{\tilde{s}}_{A,t}+{\tilde{f}}_{A,t}}+\left(1-p_{A,t}\right)\cdot \frac{{\tilde{s}}_{B,t}}{{\tilde{s}}_{B,t}+{\tilde{f}}_{B,t}},$$

and analogously for action 2. Moreover, *Q* (***z***) is the reward, which is set to 0 except for the undesirable states identified by the degree of constraining, in which case it equals the negative penalty. If we are at the end of the horizon, i.e. *t* = *T*, we do not make any patient randomisation decision, and so the above simplifies to *ℱ*_*T*_(***z***) = Q (***z***).

The ultimate optimisation problem is to find the randomisation procedure which maximises the expected total reward over the horizon for a given prior distribution at time *t* = 0, namely, *ℱ*_0_(*s*_*A*,0_, *f*_*A*,0_, *s*_*B*,0_, *f*_*B*,0_). Analogously to the definition of *ℱ*_*t*_ (***z***) in (1), we can define the optimal action *a*_*t*_ (***z***) as the action at time *t* that achieves the maximum in (1).

This is a generalisation of the two-armed finite-horizon Bayesian Beta-Bernoulli bandit problem which can still be solved exactly, in an analogous way to the original variant, using DP methods (specifically, a backward recursion algorithm) to give an optimal RAR procedure. For details of the backward recursion algorithm, refer to Williamson et al. (2017, Appendix). The backward recursion algorithm for the generalised CRDP variant is outlined in Appendix A.1. Note that this framework can be applied more generally to other objective functions, depending on the goal of the trial (e.g. Pertile et al., 2014; Alban et al., 2018; Chick et al., 2017, 2020).

### 2.3 Alternative interpretation of CRDP

**Fig. 1:**
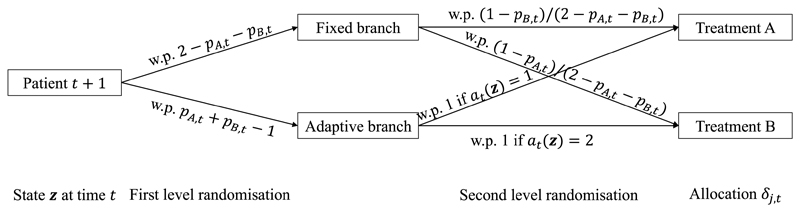
Alternative representation of the CRDP randomisation procedure.

Now we develop an alternative interpretation of the CRDP randomisation procedure, which translates to a bi-level randomisation of every patient. Imagine that there are two parallel branches of the trial: a *fixed branch* and an *adaptive branch* (see the probability tree in Fig. 1). In the first level, each patient *t* is randomised between these two branches, that is, either routed to the fixed branch with probability 2 − *p*_*A*,*t*_ − *p*_*B*,*t*_ or to the adaptive branch with the complementary probability *p*_*A*,*t*_ + *p*_*B*,*t*_ − 1 (this interpretation would thus require *p*_*A*,*t*_ + *p*_*B*,*t*_ ≥ 1). In the second level, if patient *t* had been routed to the fixed branch, then they will be randomised using a fixed randomisation ratio of 1 − *p*_*B*,*t*_ to arm *A* versus 1 − *p*_*A*,*t*_ to arm *B* (i.e. the second-level randomisation probability to arm *A* is (1 − *p*_*B*,*t*_)/(2 − *p*_*A*,*t*_ − *p*_*B*,*t*_)). Note that such patients are randomised between treatments using time-dependent probabilities (since *p*_*j*,*t*_’s are time-dependent in general), but these are defined before the trial and thus not response-adaptive. Alternatively, if they had been routed to the adaptive branch, then they will be allocated matching the CRDP actions to treatments directly without any further randomisation. That is, under action 1, patient *t* + 1 is allocated to treatment *A* with probability 1 and to treatment *B* with probability 0; under action 2, patient *t* + 1 is allocated to treatment *B* with probability 1 and to treatment *A* with probability 0 (or equally randomised between these two arms if the two actions lead to the same objective value). Note that such patients are allocated in a response-adaptive way, as the action depends on joint data ***z*** available at time *t.*

Note that if *p*_*A*,*t*_ = *p*_*B*,*t*_, then the fixed branch randomises uniformly between the arms (as in equal fixed randomisation). Similar randomisation procedures have been proposed and studied previously. One of the pioneering, and still commonly used, algorithms in reinforcement learning is the so-called *epsilon-greedy* algorithm, which aims to soften the greedy algorithm (of always allocating to the arm with the highest value of some measure) by forced exploration with a small probability (epsilon) by randomly choosing an arm uniformly. This name was coined by Sutton (1996), although the algorithm was introduced in Thrun (1992a,b) as an exploration technique under the name *semi-uniform distributed exploration* and is also a typical feature of the Q-learning algorithm (Watkins, 1989, p. 178). While the epsilon-greedy algorithm uses a constant epsilon (typically *ϵ* = 0.1), other variants have been proposed in the literature. These include the *epsilon-decreasing* algorithm, in which the exploration probability decreases over time in a predefined way, and the *adaptive epsilon-greedy* algorithm, in which the exploration probability is dynamically adapted to accumulating observations (Sutton and Barto, 2018). In these variants, the second branch is typically taken as myopically optimal, which corresponds to the special case of CRDP with horizon *T* = 1.

### 2.4 Benchmark randomisation procedures

The *fixed randomised* procedure randomises patients to treatment *A* with a fixed probability and to treatment *B* with the complementary probability. In a two-armed trial, this probability is often 50%, corresponding to a 1:1 randomisation ratio, but in general it can be any value strictly between 0 and 1. This procedure is very common in practice and will act as a reference to which the RAR procedures will be compared against.

We will also compare the procedures to the *delayed randomised play-the-winner rule* (DRPWR) which is the rule most often suggested for delayed response settings (Hardwick et al., 2006). The randomised play-the-winner rule (RPWR), proposed by Wei and Durham (1978), is a type of RAR procedure which uses all of the past allocations and responses to influence the allocation decision of the next patient. It can be represented by an urn model whereby the urn accumulates more balls representing the superior treatment, thus increasing the probability that a patient will be allocated to the current best treatment. Delayed responses can be accommodated by simply updating the urn composition when the responses become available. However, this would slow the adaptation and reduce the benefit to patients, particularly those recruited early (Rosenberger, 1999). Two different DRPWR models are discussed in the literature (see e.g. Atkinson and Biswas, 2014, Chapter 3) which we briefly summarise. First, Wei (1988) introduces another set of indicator variables *ϵ*_*j*,*i*+1_ (in addition to the treatment allocation and response indicators of the RPWR), which determine whether or not a previous patient’s response has been observed before allocation of the next patient, i.e. *ϵ*_*j*,*i*+1_ = 1 if the response of the *j*th patient is observed before entry of patient *i* + 1 (*j* = 1, …, *i*), and 0 otherwise (Biswas, 2003). Thus, the conditional probability that patient *i* + 1 is allocated to treatment *A* given all of the previous allocations *δ*_*j*_, responses *y_j_* and response statuses *ϵ*_*j*,*i*+1_ for *j* = 1, …, *i* is

(2) $$\begin{array}{l} P\left(\delta_{i+1}=1|\delta_{1},\ldots ,\delta_{i},y_{1},\ldots ,y_{i},\epsilon_{1}{,}_{i+1},\ldots ,\epsilon_{i}{,}_{i+1}\right)\\ \;=\frac{\left\{\alpha +\beta \left(2\sum_{j=1}^{i}\epsilon_{j,i+1}\delta_{j}y_{j}+\sum_{j=1}^{i}\epsilon_{j,i+1}-\sum_{j=1}^{i}\epsilon_{j,i+1}\delta_{j}-\sum_{j=1}^{i}\epsilon_{j,i+1}y_{j}\right)\right\}}{2\alpha +\beta \sum_{j=1}^{i}\epsilon_{j,i+1}}, \end{array}$$

where *α* is the initial number of each type of ball in the urn and *β* is the current number of type *A* balls that have been added to the urn (following each success on treatment *A* and each failure on treatment *B*). The numerator is the current number of type *A* balls in the urn and the denominator is the total number of balls in the urn at this point. Bandyopadhyay and Biswas (1996) introduce a second model with a slight modification that ensures the denominator of the conditional allocation probability in (2) is free of any random variables. Biswas (1999) compares these two models showing that they are asymptotically equivalent and that there is no significant difference between their performances. Hence, we consider the first version of the DRPWR as a comparator in this paper.

### 2.5 Performance measures

Although CRDP is set within a Bayesian framework, we use simulation to evaluate its performance according to standard frequentist criteria, which is essential in practice for regulatory purposes (Zhang et al., 2019). In the subsequent sections, we consider the following hypothesis for a two-armed trial: *H*_0_ : *θ_A_* = *θ_B_* versus *H*_1_: *θ_A_* ≠ *θ_B_*, which we test using Fisher’s exact test with a nominal significance level of 0.1.

Simulating the trial under the assumption of a treatment difference and calculating the proportion of times *H*_0_ is correctly rejected in favour of *H*_1_ gives an estimate of power. Conversely, when simulating under the null hypothesis of no treatment difference, the proportion of times *H*_0_ is incorrectly rejected corresponds to the estimated type I error. We also report the average bias and mean squared error of the treatment effect estimator under both hypotheses. In terms of evaluating the in-trial patient benefit, we focus on the percentage of patients allocated to the superior treatment. Note that under *H*_0_, the superior treatment is taken to be the control arm.

Throughout, we focus on the scenarios in which θ_*A*_ = 0.5, *θ_B_* ∈ {0.1, 0.2, …, 0.9} and *n* = 75 so results are consistent with, and comparable to, those reported in Williamson et al. (2017). The observed patterns and conclusions remain the same for other values of *θ_A_* so we do not report them here.

## 3 The effect of delayed responses on (CR)DP

The CRDP and the DP procedures are jointly abbreviated as (CR)DP from hereon. In this section, we consider the CRDP procedure with tuning parameters as suggested in Williamson et al. (2017), that is, *T* = *n*, *p*_*j*,*t*_ = 0.9 for all *j*, *t* and *ℓ* = 0.15*n* with penalty −*n*, which produce a good balance between power and patient benefit across a wide range of scenarios and samples. The DP procedure is defined by taking *T* = *n*, *p*_*j*,*t*_ 1 = 1 for all *j*, *t* and *ℓ* = 0. In the case of no delay, (CR)DP randomises the first patient using equal fixed randomisation because the prior distributions on both arms are the same. Applying the same model when responses are delayed means that equal fixed randomisation is used to allocate patients until the first response is observed. After all patients have been allocated, we assume that all patient outcomes will be available, and thus will contribute to the estimated treatment effect at the end of the study.

In order to explore the impact of delayed responses when applying (CR)DP, we use simulation to evaluate its performance in a range of scenarios for different delay lengths. By first understanding the impact of a delayed response, we can then take steps to modify the procedure in Section 4. Moreover, as Wason et al. (2019) pointed out, “it is important that theoretical work that proposes and promotes adaptive designs clearly lays out any reduction in their reported efficiency benefits when there is substantial delay in outcome evaluation”.

### 3.1 Fixed delays

We first focus on a *deterministic* (or fixed) delayed response model which assumes that there is a constant time between allocations and a fixed delay of length *d* > 0 between allocating a patient to a treatment and observing their outcome. As a result, we will know exactly how many patients are in the pipeline at each stage in the trial which, for *t* ∈ {*d* + 1, …, *n*}, will remain of fixed length equal to *d*. Although a patient response may occur at any time, in binary response trials (considered in this paper), interest is only in if it has occurred by the specified follow-up time. If the response has not occurred by the time of follow-up (which could be due to patient dropout), this is typically reported as a treatment failure in binary response trials.

**Fig. 2:**
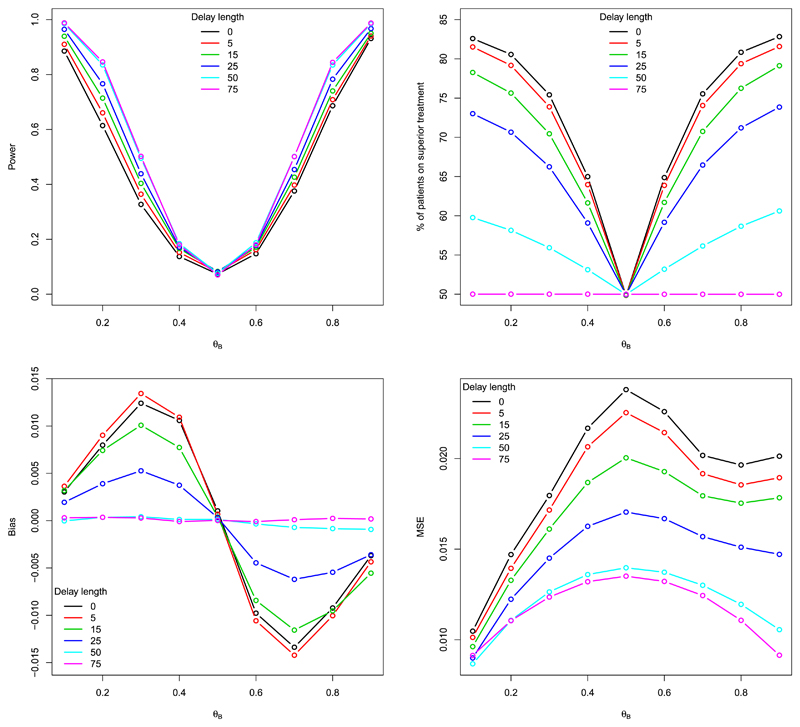
Power/type I error, % of patients on the superior treatment, the average bias and MSE of the treatment effect estimator for CRDP when *n* = 75, *θ_A_* = 0.5 and *θ_B_* ∈ (0.1, 0.9) for different fixed delay lengths (estimated over 100,000 simulations). (For interpretation of the colours in the figure(s), the reader is referred to the web version of this article).

The results are illustrated for *d* = 0, 25, 50, 75, and also for 5 and 15 since more interest is in what happens for shorter delays, as this is where the most marked changes in performance of these procedures occur. The reason for including the results for no delay is so we can clearly evaluate how the delayed responses are affecting the performance measures relative to the base case. Further, recall that *d* = 75 corresponds to fixed, equal randomisation. The results illustrated in Fig. 2 correspond to changes in the performance of the CRDP procedure, and analogous results for the DP procedure are displayed in Fig. A.8 of Appendix A. We include results for the DP procedure to show how the delay affects the procedure in the absence of the randomisation and constraining.

**Power.** The top left plot in Fig. 2 illustrates the changes in statistical power for CRDP, with the results for *θ_A_* = *θ_B_* corresponding to the type I error. The most notable observation is that the power *increases* with delay length. This because, as the length of the delay increases, the adaptation is slowed and the procedure approaches that of fixed randomisation meaning there is less imbalance between the treatment arms. However, the observed changes in power are not linear, but rather seem like following the law of diminishing returns. For example, the increase in power from *d* = 0 to *d* = 5 is approximately the same as from *d* = 5 to *d* = 15, with negligible changes as the delay length increases from 50 to 75. The expected patterns, such as the power increasing with the size of the treatment difference, are evident for all delay lengths. In terms of the type I error rates, they are well controlled at the desired 0.10 level for all delay lengths.

**Patient benefit.** The top right plot in Fig. 2 illustrates the changes in the percentage of patients allocated to the superior treatment, i.e. the patient benefit, for CRDP. When *θ_A_* = *θ_B_*, the procedure allocates approximately 50% of patients to the superior treatment whatever the delay length, as expected. In general, we observe that the number of patients in the trial receiving the superior treatment *decreases* as the delay length increases because a longer delay means a longer period of equal randomisation at the start of the trial.

Consider the scenario in which *θ_A_* = 0.5 and *θ_B_* 0.1. For the case of no delay, approximately 83% of patients in the trial are allocated to the superior treatment and for a delay of length 25, approximately 73% of patients are allocated to the superior treatment. Thus, we only lose approximately 10% of the patient benefit in this case. Furthermore, compared to fixed randomisation (illustrated by the pink line in Fig. 2), the gain in patient benefit remains high. Even for a delay length of 50 (two thirds of the trial size), there are still worthwhile gains in terms of patient benefit of implementing CRDP, with approximately 10% more patients being allocated to the superior treatment relative to fixed randomisation.

It is also clear from the plots that as the magnitude of the treatment difference increases (i.e. as *θ_B_* decreases from 0.5 to 0.1 or increases from 0.5 to 0.9 for *θ_A_* = 0.5), the percentage of patients allocated to the superior arm also increases across all delay lengths less than 75, as expected.

**Bias.** The bottom left plot of Fig. 2 shows the changes in the average bias of the treatment effect estimator $\hat{\Delta }={\hat{\theta }}_{A}-{\hat{\theta }}_{B}$ (where ${\hat{\theta }}_{A}$ = *S*_*A*,*n*_/*N_A_* and ${\hat{\theta }}_{B}$ *S*_*B*,*n*_/*N_B_* are the observed proportions of successes on treatment *A* and *B*, respectively, by the end of the trial). We observe that, in general, the bias *decreases* as the delay length increases (with some slight discrepancy for delay lengths of 0 and 5). The decrease in bias is due to the values of *s*_*A*,*n*_, *s*_*B*,*n*_, *N*_*A*_ and *N*_*B*_ varying with delay length. As an example, consider the scenario in which *θ_A_* = 0.5 and *θ_B_* = 0.1. For shorter delays, there will be fewer patients allocated to the inferior treatment (arm *B*) so that *N_*B*_ < *N*_A_*. As a result, ${\hat{\theta }}_{B}$ will be underestimated, which is shown in Williamson et al. (2017), so the treatment effect estimator, $\hat{\Delta }$ will be larger, leading to a larger bias. Alternatively, as *d* → 75, then *N*_*B*_ → *N*_*A*_ until eventually *N*_*B*_ ≈ *N*_*A*_ when *d* = 75. Therefore, ${\hat{\theta }}_{A}$ and ${\hat{\theta }}_{B}$ will be closer to their true values, hence giving rise to a smaller bias. Note that it will be useful to refer to the raw estimates of *θ_A_* and *θ_B_* in Table 1 to illustrate this.

**Mean squared error.** The bottom right plot in Fig. 2 shows that the mean squared error (MSE) of the treatment effect estimator *decreases* as the delay length increases across all scenarios. Since the MSE is a function of the bias, this could simply be attributable to the observed decrease in bias with delay. However, after plotting the variances of the treatment effect estimator (not included here), which follow exactly the same pattern as the MSE plots, this confirms that the variability of the estimator does indeed decrease with delay.

#### 3.1.1 Comparison of (CR)DP to DRPWR

**Fig. 3:**
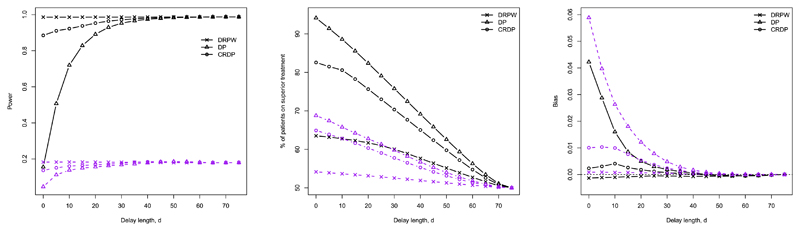
The changes in power, % of patients on the superior treatment and the average bias of the treatment effect estimator for (CR)DP and DRPWR as the length of the fixed delay increases when *n* = 75, *θ_A_*, *θ_B_*) = (0.5, 0.1) (black line), and *θ_A_*, *θ_A_* = (0.5, 0.4) (purple line) (estimated over 100,000 simulations). (For interpretation of the colours in the figure(s), the reader is referred to the web version of this article.)

In this section, we explore how the DRPWR (described in subsection 2.4) compares to the (CR)DP procedures for a range of delay lengths via simulation. In the following, we consider a scenario in which there is a treatment difference, but the results for no treatment difference are shown in Fig. A.12 of Appendix A. In particular, we focus on the case in which *θ_A_* = 0.5 and *θ_B_* = 0.1 (represented by the black lines in Fig. 3). However, we have also added the results corresponding to *θ_A_* = 0.5 and *θ_B_* = 0.4 (represented by the purple lines in Fig. 3) to show that similar trends are observed for smaller treatment differences. Plots showing the performance of DRPWR over a wider range of scenarios are also provided in Fig. A.10 of Appendix A.

**Power.** The first plot in Fig. 3 illustrates the changes in power as the delay length, *d*, increases. The power of (CR)DP is shown to increase hyperbolically, with the largest changes occurring for shorter delay lengths and practically no change occurring as *d* increases from 40 to 75. In contrast, the power of the DRPWR remains fairly constant for all delay lengths. The power of the RPWR is already high when there is no delay because it does not create enough imbalance between the two treatments, and thus there is little room for improvement.

Comparing the procedures, although the DRPWR attains the highest power for delays up to around 45 (at which point the procedures essentially converge), CRDP also performs very well (even for small delays), whereas the power of DP is insufficient and lies below 80% for delays up to length 15. For example, when the delay is 5, the power of DRPWR and CRDP is above 90% but for DP, it is close to 50%.

**Patient benefit.** The second plot in Fig. 3 shows how the percentage of patients allocated to the superior treatment varies as *d* increases. Similarly to the (CR)DP, as the delay length increases, the DRPWR allocates fewer patients to the superior arm. For DP, the percentage of patients allocated to the superior treatment decreases linearly at a relatively constant rate compared to the CRDP which decreases at a slower rate, and the DRPWR which decreases even slower. Further, (CR)DP allocates substantially more patients to the superior treatment than the DRPWR, most markedly for shorter delay lengths. For example, Fig. 3 shows that when *d* = 5, DP and CRDP allocate approximately 91% and 81% of patients to the superior arm, respectively, while DRPWR allocates 63%. Even when there is a smaller treatment difference (as shown by the purple lines), CRDP continues to allocate more patients to the superior treatment. In this case, when *d* = 5, CRDP allocates approximately 64% of patients to the superior arm, while DRPWR allocates 54%.

**Bias.** The third plot in Fig. 3 illustrates the changes in the bias of the treatment effect estimator as *d* varies. We have already identified that, generally, the bias of (CR)DP decreases with delay and occurs at a much quicker rate for DP. In contrast, the bias values following the DRPWR appear to be fairly robust to changes in delay, remaining close to 0 for all delay lengths, with a very slight decrease as *d* increases. Note that the scale of this plot is very small so the differences observed are only negligible (to three or four decimal places).

### 3.2 Random delays

The assumption of a fixed delay, considered above, leads to a simple model which allows fundamental insights to be made, but may not provide an acceptably good approximation for many clinical trials in which there is randomness in patient arrivals. Therefore, in this section, we consider a simple stochastic model in which patients now arrive randomly and, consequently, the number of patients in the pipeline at any stage of the trial is also random. This formulation is equivalent to assuming deterministically regular arrivals with a random response time. Since it is more intuitive to interpret random delay as the random time from allocation to response (rather than the random number of patients in the pipeline), we use this context to illustrate the effect of random delays on (CR)DP without loss of generality. This is the set-up also used in Hardwick et al. (2006). However, this is purely for ease of interpretation and, in clinical trial practice, it is not typical to have a binary endpoint that is observed with a random delay.

We use a Bernoulli random variable with probability *r* to determine which patients in the pipeline have responded at each stage *t* in the trial. This is equivalent to assuming a geometric response time (or delay length), *Y_i_* ~ Geometric(*r*) for each patient *i* = 1, …, *n*, i.e. the number of time units (e.g. days) before response. If a patient has responded, we record their observation, update the states accordingly and remove this patient from the pipeline. Otherwise, if the patient has not yet responded, they remain in the pipeline and we simply proceed to allocate the subsequent patient based on whatever information is currently available. As in the fixed delay setting, we assume complete data at the end of the study.

We vary the response probability *r*, i.e. the probability of a patient responding at each stage, to explore the impact of random delays on (CR)DP. So that the results are presented similarly to those in the previous section, we illustrate the performance measures for different *expected* delay lengths, taking values of *r* = 1/(1 + 𝔼(*Y_i_*)) such that 𝔼(*Y_i_*) = 0, 5, 15, 25, 50, 75 and 100 for each *i* (note that, in this case, it is possible to have 𝔼(*Y_j_*) > *n* and equal fixed randomised procedure would be recovered by 𝔼(*Y_i_*) → ∞).

Since the expected value of a geometric random variable *Y_i_* is given by 𝔼(*Y_i_*) = (1 − *r*)/*r*, to do this, we will choose values of *r* = 1/(1 + 𝔼(*Y*_*i*_)) such that 𝔼(*Y*_*i*_) = 0, 5, 15, 25, 50, 75 and 100 for each *i*. Note that we include an expected delay length of 100 here to demonstrate that, in the random delay case, the (CR)DP gives rise to different performance measures for expected delays greater than the trial size of 75. This is in contrast to the fixed delay case in which, for all delays ≥ 75, (CR)DP mimics equal randomisation.

**Fig. 4:**
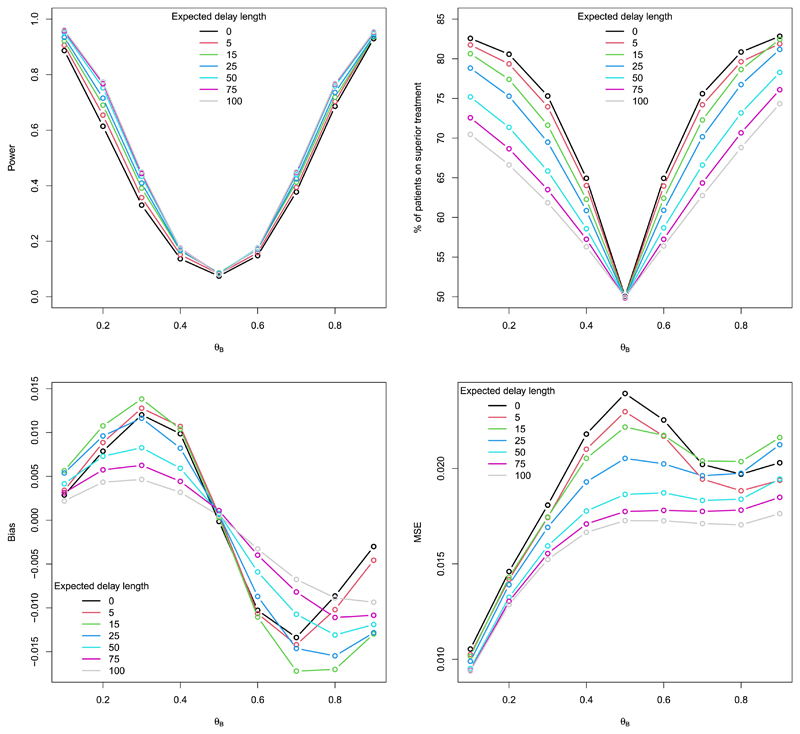
Power/type I error, % of patients on the superior treatment, the average bias and MSE of the treatment effect estimator for CRDP when *n* = 75, *θ_A_* = 0.5 and *θ_B_* e (0.1,0.9) for different expected random delay lengths (estimated over 100,000 simulations). (For interpretation of the colours in the figure(s), the reader is referred to the web version of this article.)

Fig. 4 is the analogue of Fig. 2 but for the random delay case. The overall trends observed in the performance measures as the expected delay lengths increase are similar to those for the fixed delay case. However, there are some immediate differences (see Fig. A.14 in Appendix A). In particular, the top right plot of Fig. 4 shows that the percentage of patients allocated to the superior treatment appears to be larger for the random delay case. The bias and MSE values are also larger when the delay is random, and there is little difference in the power as the expected delay length increases. These observations are due to a mixture of reporting averages and the fact that there is inherent variability in the results that goes beyond that of simulation error, owing to the underlying random nature of the delay (see Appendix A.5 for further details).

The corresponding plot illustrating the effect of a random delay on the performance of DP is shown in Fig. A.9 of Appendix A.

#### 3.2.1 Comparison of (CR)DP to DRPWR

We now compare the performance of the (CR)DP in trials with a random delay to the DRPWR. In the following, we consider how the performance measures vary with the *expected* delay length for a treatment difference, where *θ_A_* = 0.5 and *θ_B_* = 0.1. Corresponding results for no treatment difference are shown in Fig. A.13 of Appendix A. For an alternative illustration of how the DRPWR (with random delay) behaves for a wider range of scenarios under different expected delay lengths, see Fig. A.11 in Appendix A.

**Fig. 5:**
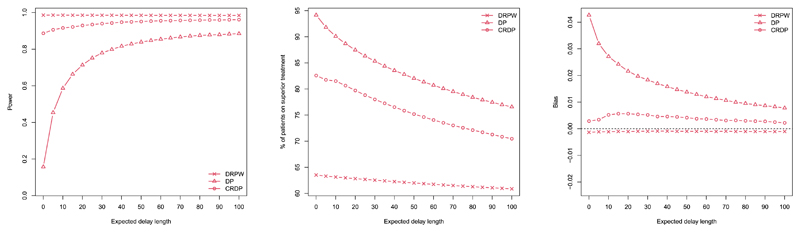
The changes in power, % of patients on the superior treatment and the average bias of the treatment effect estimator for (CR)DP and DRPWR as the expected delay length increases, when *n* = 75, *θ_A_* = 0.5 and *θ_B_* = 0.1 (estimated over 100,000 simulations).

**Power.** The first plot in Fig. 5 shows the changes in power for the (CR)DP and DRPWR as the expected delay length increases. As in the fixed delay case, the greatest changes in power for the (CR)DP procedures occur for shorter expected delay lengths. For CRDP, the power remains constant for delays expected to be greater than 65, but for DP it continues to increase. The power of DRPWR, on the other hand, remains relatively stable for all expected delay lengths and attains values very close to those obtained when there is a fixed delay.

Relative to DRPWR, the (CR)DP procedures have smaller power for all expected delay lengths. Again, this difference is much more prominent for DP. For example, when the delay length is expected to be 5, the power of DRPWR is 0.99, that of CRDP is 0.91 and that of DP is 0.46. For expected delays over 40, the difference in power between DRPWR and CRDP is at most 0.03.

**Patient benefit.** The second plot in Fig. 5 compares how the percentage of patients allocated to the superior treatment varies as the expected delay length increases from 0 to 100 for the (CR)DP and DRPWR. (CR)DP continues to maintain important levels of patient benefit (around 77% and 67% for DP and CRDP, respectively) even for an expected delay of 100. The DRPWR starts with a small patient benefit (around 64%) and only decreases by a small amount (to around 61%) as the expected delay increases. Moreover, the rate of decrease for these procedures remains relatively constant. Compared to DRPWR, (CR)DP allocates significantly more patients to the superior treatment for all expected delay lengths considered. In particular, for an expected delay length of 5, (CR)DP and DRPWR allocate approximately the same number of patients to the superior arm as we observed in the fixed delay case.

**Bias.** The third plot in Fig. 5 illustrates the changes in the average bias of the treatment effect estimator as the expected delay length varies. Overall, for the (CR)DP procedure, the trend in bias appears to be decreasing, which is much more apparent for DP. The bias values corresponding to DRPWR do not change much with the expected delay and lie slightly closer to 0 than CRDP for all expected delay lengths. However, the scale of this plot is very small so the differences in the bias between DRPWR and CRDP are trivial. DRPWR and CRDP consistently outperform DP, but the differences are considerably greater for shorter expected delays. For example, when the expected delay length is 5, the bias of DP is ten times larger than that of CRDP.

## 4 Adjusting the time horizon of (CR)DP for fixed delays

As we have seen above, (CR)DP already performs relatively well in the presence of delayed responses with slight gains in power and a loss in patient benefit as the delay length increases. However, the actions are computed assuming that all *n* of them will be implemented which, due to the delay, is not the case. As a result, the optimised procedure breaks down if (CR)DP is used naively as in the previous section. Ideally, we want to retain their optimisation features as much as possible, which we address in this section. Throughout this section, we assume that there is a fixed number of patients in the pipeline.

In Section 3, the time horizon used in the MDP formulation of the (CR)DP procedure was of size *T* = *n*, i.e. the number of patients in the trial. However, when we implement this procedure with a fixed delay of length *d*, the state representing the number of unobserved patients remaining in the trial will stay the same for the first *d* patients because no observations accrue during this stage. Therefore, these patients are simply randomised (with equal probability) between the treatments, giving rise to an initial equal randomisation phase. It is only once we begin to receive observations, i.e. from time *d* + 1 onwards, that (CR)DP allocates patients *adaptively*. Importantly, the last *d* planned actions of the (CR)DP procedure are not effectuated, and thus the procedure may not achieve the objective it is optimised for, especially for CRDP which may result in the undesirable (penalised) end-of-trial states not being avoided and thus the desired constraint not being satisfied. This suggests that for a trial of size *n*, it may be more appropriate to use (CR)DP to optimise the allocation of patients *d* + 1 to *n* only, that is, for *n* − *d* of the allocation decisions, and to define and penalise the undesirable states at stage *n* − *d* rather than at stage *n* so that the desired constraint continues to be satisfied even in the presence of delayed responses. Consequently, by setting *T* = *n* − *d* in equation (1), the value-to-go function, we implement (CR)DP with a delay-adjusted time horizon (TH), which we refer to as the CRDP-TH procedure. Not only does this mean that we generate a smaller array of optimal actions, which is computationally quicker and requires less memory, but this will allow us to understand whether there are any non-negligible gains when optimising over the smallest possible time horizon instead. It will also ensure that after all *n* patients have been allocated, the desired degree of constraining will still be satisfied. Although we adjust the horizon *T*, for comparison purposes, we keep the original randomisation and constraining parameters as used in CRDP, i.e. *p* = 0.9 and *l* = 0.15*n* with a penalty of −*n*, without accounting for the observations that will be revealed after the end of the trial. We discuss this assumption at the end of this section.

**Fig. 6:**
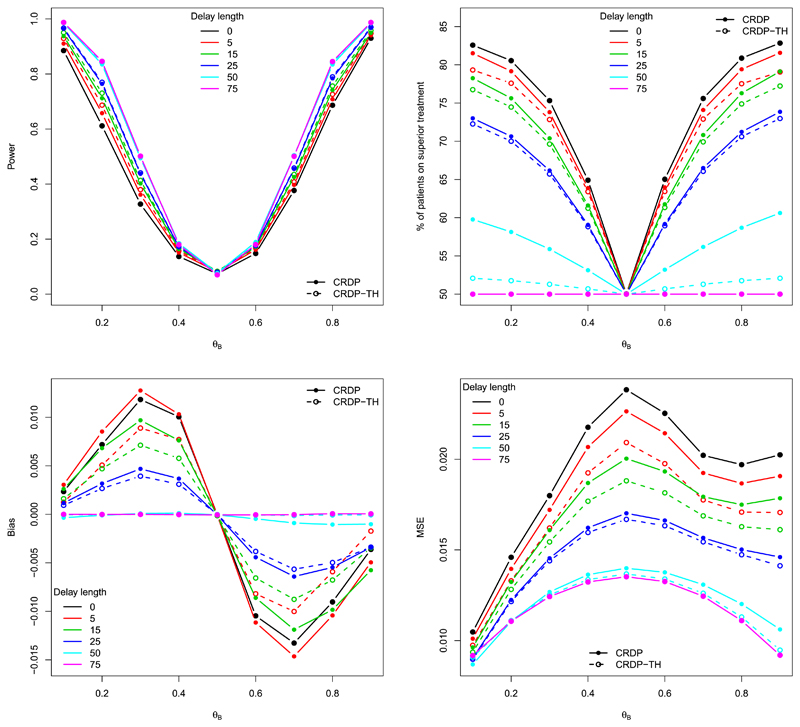
Power/type I error, % of patients on the superior treatment, the average bias and MSE of the treatment effect estimator for CRDP and CRDP-TH when *n* = 75, *θ_A_* = 0.5 and *θ_B_* ∈ (0.1, 0.9) for different delay lengths (estimated over 1, 000, 000 simulations). (For interpretation of the colours in the figure(s), the reader is referred to the web version of this article.)

Fig. 6 illustrates the performance measures of CRDP-TH (represented by the dashed lines) for a range of delay lengths. For comparative purposes, CRDP when using the original time horizon of *n* is also superimposed onto these plots (solid lines). In terms of power (top left plot in Fig. 6), there is very little difference between the two procedures, with CRDP-TH lying slightly above CRDP for shorter delay lengths since CRDP-TH is better at avoiding the undesirable states with too few observations when there is a delay. For the percentage of patients on the superior arm (top right plot in Fig. 6), the differences are more pronounced and CRDP outperforms CRDP-TH for all delay lengths (excluding 0 and 75 where both procedures are equivalent) because CRDP-TH imposes the constraints more stringently (discussed below). Further, since CRDP-TH results in less imbalance between the two treatment groups than CRDP, the corresponding bias and MSE values are also notably smaller for CRDP-TH, as illustrated in the bottom two plots of Fig. 6.

**Fig. 7:**
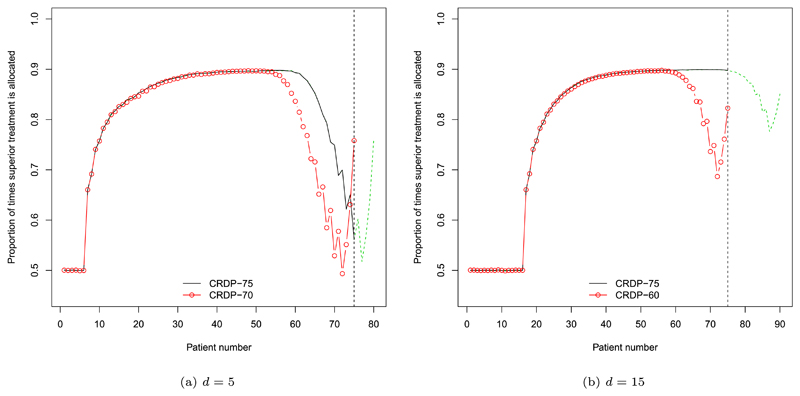
Probability of allocating a patient to the superior treatment when *θ_A_* = 0.5 and *θ_B_* = 0.9 in a trial of size *n* = 75 (estimated over 1, 000, 000 simulations). The black and red lines correspond to CRDP with time horizons *T* = *n* and *T* = *n* − *d*, respectively. The dashed green lines illustrate what the remaining *d* allocations would look like if CRDP continued. (For interpretation of the colours in the figure(s), the reader is referred to the web version of this article.)

We now discuss why CRDP is shown to attain a larger percentage of patients on the superior arm compared to CRDP-TH, with the aid of allocation plots in Fig. 7. For illustrative purposes, we will take *θ_A_* = 0.5 and *θ_B_* = 0.9 but the same reasoning also applies to other scenarios. In Williamson et al. (2017, Fig. 6), for the no delay case, the average allocation probability to the superior treatment oscillates markedly for the final 15 patients (in a trial of size 75) in order to satisfy the constraint, thus indicating that an important number of allocations to the inferior arm occur towards the end of the trial. However, when the CRDP time horizon *T* is equal to the trial size *n* and there is a delay of length *d*, the final *d* decisions are not effectuated. Consequently, this final exploration phase, which is illustrated by the dashed green lines in Fig. 7a and Fig. 7b for *d =* 5 and 15, respectively, is now ignored. Nevertheless, CRDP will continue to allocate the required number of patients, as specified by the constraint, to the inferior arm because of the unaccounted pipeline patients. In fact, on average, it will “over-satisfy” the constraint because the number of allocations made to the inferior arm during the initial equal randomisation phase (as a result of the delay) will, on average, exceed those that are no longer being made at the end. This is evident from Fig. 7a and Fig. 7b where it is clear that the proportion of times the superior (inferior) treatment is allocated during the “non-effectuated” phase in green is substantially greater (smaller) than that during the equal randomisation phase.

In contrast, by using the smallest possible time horizon of *n – d* instead, there will be even more allocations, on average, to the inferior arm because the exploration phase towards the end of the trial is still incorporated (as in the no delay case) (see the red lines in Figs. 7a and 7b). Hence, we see a smaller percentage of patients on the superior treatment, and thus higher power, for CRDP-TH with a time horizon of 75 − *d* compared to CRDP with the longer time horizon of 75.

The patient allocation plots in Fig. 7 also illustrate the effect of changing the delay length *d* on the average allocation probabilities when using CRDP and CRDP-TH. For example, the black line in Fig. 7a shows the average allocation probability to the superior treatment under the CRDP procedure with time horizon equal to the trial size *T* = 75, a fixed delay of *d* = 5 and a degree of constraining equal to 15% of the total sample size (i.e. approximately 12 patients on each arm). We see that near the end of the trial, by around patient number 60, the proportion of times the superior treatment is allocated decreases in order to satisfy the constraint. However, when the delay length is increased to *d* = 15, Fig. 7b shows that there is no longer this decrease near the end of the trial because, in this case, it is likely that the minimum sampling requirement on each arm will have already been fulfilled (owing to the longer delay length and, consequently, the longer initial equal randomisation phase). The plots for CRDP-TH (in red) similarly show that as the delay length increases, the need to allocate as many patients to the inferior treatment at the end of the trial is reduced.

It is not obvious whether the observed differences in Fig. 6 are due to the change in time horizon, or the fact that CRDP-TH is effectively satisfying a stricter constraint. To isolate the impact of the time horizon alone on the performance of the procedure, we remove the constraint and randomisation from the procedure, and revert back to the original DP procedure. The corresponding performance measures illustrated in Fig. A.16 and allocation plots in Fig. A.17 of Appendix A.6 indicate that DP and DP-TH behave similarly.

## 5 Discussion

In this paper, we have developed a generalisation of the recently proposed CRDP procedure, which allows for additional flexibility to adjust to different practicalities of clinical trials and sequential experiments more generally. A novel representation of CRDP, depicted via a probability tree, was also provided to aid interpretation and show that it can be viewed as a non-myopic generalisation of the epsilon-greedy algorithm, which is well-known in the reinforcement learning literature.

We then evaluated how the (CR)DP procedure performs when responses are observed after a delay, which is an important – and commonly asked – question in practice. In Section 3, we demonstrated that we gain slightly in terms of power and bias through the delay (so, from the statistical perspective, delay could be viewed as a positive attribute), but we lose in terms of patient benefit (which is the main advantage of using such RAR procedures over alternatives). However, this loss is not overly concerning: e.g. for a relatively large fixed delay length which is one third of the sample size 75, the percentage of patients on the superior treatment when *θ_A_* = 0.5 and *θ_B_* = 0.1 is approximately 23% higher for CRDP and 30% higher for DP than the traditional approach of equal randomisation. Even for a fixed delay of length 50, there are still worthwhile patient benefit gains, while the losses in power, bias and MSE are minimal. Only when the fixed delay length is greater than or equal to the trial size do the gains fully disappear; for random delays, patient benefit gains persist even in this case. As such, our results partly contradict the common opinion that “adaptive allocation has no benefit when there are long delays” (Berry and Stangl, 1996, Chapter 4).

Further, when compared to the performance of the most commonly studied rule for delayed response scenarios, namely the DRPWR (Hardwick et al., 2006), there are considerable improvements with respect to the patient benefit for (CR)DP. Therefore, this evaluation has shown that the (CR)DP procedures already perform well in trials with delayed responses since they continue to maintain their patient benefit advantages over other procedures for a range of (expected) delay lengths. More specifically, for short to moderate delays, (CR)DP incurs only a slight loss in patient benefit (relative to the no delay case), which reflects what has been found in both the statistical and bandit literature for other response-adaptive procedures (Kaibel and Biemann, 2021). Thus, the main message to convey is that (CR)DP is fairly robust to delays, whether fixed or random.

The next part of this paper suggested an approach, based on adjusting the time horizon of the corresponding MDP, to account for a fixed delay. Investigation of this approach illustrated the underlying interdependence between the delay length and constraint. In particular, if the delay length already satisfies the desired constraint via the initial equal randomisation phase of the first *d* + 1 patients, then it may not be necessary to adjust the time horizon of (CR)DP. However, in general, (CR)DP with a non-adjusted time horizon may not even reach the final stage where the constraints are specified and thus, adjusting the time horizon is likely to be a preferred approach. An interesting topic for further research is how to appropriately tailor the degree of constraining within the CRDP formulation according to the different delay lengths. One way to achieve this for the CRDP-TH procedure is to subtract the expected number of patients that are on the inferior arm during the final *d* allocations from the current degree of constraining. These translate to observations which the current procedure is “blind” to because they only become available after all allocations have been made, hence why the constraint may end up being stricter than desired.

This research provides impetus for several other areas of further work, some of which will now be outlined.

The simulation study considered is restricted to the setting of a two-arm trial with binary endpoints and a simple stochastic mechanism using a geometric distribution for the discrete time to model the delay. Extending this to other practical settings would provide valuable insight into how CRDP performs more generally. Moreover, since CRDP was developed in the context of rare diseases, the focus has been on relatively small trial sizes. In this paper, the code written in the programming language R from Williamson et al. (2017) was used for all computations, which reported that (CR)DP could be implemented for trial sizes of up to 215 on a standard computer with 16 GB RAM. However, Jacko (2019b) showed that DP solutions are tractable for much larger horizons than are commonly believed. If using another programming language, such as Julia, and a more effective coding syntax (see Jacko, 2019a), a computer with 32 GB RAM can solve the two-armed bandit problem for a trial size of up to 1440 and 4440, depending on whether storage of the optimal allocation policy is or is not required, respectively.

Development of a randomisation procedure analogous to (CR)DP for the non-binary case is theoretically possible, but will become computationally infeasible for much smaller trial sizes than for the current binary response variant. The trialist could, however, still employ the binary-response (CR)DP by dichotomising the primary endpoint, which is a widely adopted approach in clinical research (Royston et al., 2006), or by using an auxiliary endpoint correlated with the primary endpoint. Although Williamson and Villar (2020) showed that dichotomisation can reduce the patient benefit of RAR procedures compared to when using the original endpoint, if meaningfully defined, this loss may only be negligible and will still bring important patient benefit gains over alternative RAR procedures. Moreover, the allocation procedure could dichotomise but the final analysis could use the continuous endpoint, which may reduce the loss in efficiency. Another option is to adjust the degree of randomisation in order to reflect the trialist's confidence in the correlation between the primary and auxiliary endpoint.

Similarly for extending (CR)DP to trials involving more than two arms; although possible in theory, it will soon become computationally challenging in practice. Alternatives which closely approximate the DP procedure are the Whittle index and the Gittins index (see e.g. Villar et al., 2015a; Jacko, 2019b; Williamson and Villar, 2020). However, these are yet to be modified to include constraints, as in the CRDP procedure. This may not always be possible, especially for constraints depending on more than one arm, because the Whittle and Gittins indices function by decomposing the trial-level optimisation problem into single-arm optimisation subproblems. Nevertheless, single-arm constraints, such as the number of observations from each arm as considered here, should be implementable. Moreover, if constraints are not required, then the degree of randomisation can be implemented easily using the Whittle or Gittins index, instead of the DP procedure, in the alternative interpretation described in subsection 2.3. The concepts introduced in this paper, namely, adjusting the time horizon according to the delay length, can also be applied to the Whittle index policy, as well as any other time dependent approach more generally.

(CR)DP was formulated under the assumption that patients allocated to the same treatment will have the same expected response. However, in practice this may be unreasonable if there are certain covariates which influence their response. For example, the success of a cancer treatment may depend on whether the patient is a smoker, in which case, only a subset of the available responses may be relevant in determining the current patient's randomisation probability. Incorporating covariates into such designs forms another area of future work where application of the index policies would be better suited than the DP techniques considered here (e.g. Villar and Rosenberger, 2018) to circumvent the curse of dimensionality as the state space grows.

Similar to previous contributions (such as Zhang et al., 2019), we have assumed an absence of time trends caused by a change in patient characteristics during the recruitment phase, such as the most severely ill patients entering the trial as soon as possible. This possibility of so-called patient (or population) drift is a major criticism of RAR in general (see e.g. Rosenberger et al., 2012, Section 4.3) since it can lead to biased parameter estimates. One solution is to use covariate-adjusted RAR if the underlying covariates causing the heterogeneity are known in advance. Examples of recent developments in this area include Villar and Rosenberger (2018) and Villar et al. (2018). Alternatively, one may consider using block RAR to reduce the bias caused by patient drift (see e.g. Magirr, 2011).

Accrual (or selection) bias may also contribute to heterogeneity in patient recruitment over time. For example, with CRDP, patients may prefer to enter the trial earlier since, as we have seen in Section 4, patients entering the trial later may be more likely randomised to the inferior treatment in order to satisfy the constraint specified by CRDP. However, typically in response-adaptive trials, it is more desirable for patients to enter the trial later because, that way, their probability of being randomised to the better treatment will be higher. This highlights that, regardless of the constraint, this type of bias still poses a problem. The introduction of the constraint in CRDP may even circumvent, or at least mitigate, the effect of accrual bias since there is no longer an obvious “desirable” stage at which to enter the trial. It may even be the case that patients refuse to be allocated to a particular treatment or drop out, resulting in fewer patients on one arm. However, this is a concern in all studies and is typically mitigated through blinding and intention-to-treat analysis.

All the results presented in this paper assume a uniform prior for the unknown success probabilities of each arm. However, one could also consider an informative prior based on data from previous trials or expert opinion (Dallow et al., 2018; Williams et al., 2021), for example. The (CR)DP procedure also allows for implementing a decreasingly informative prior (see Donahue and Sabo, 2021) by modifying the rewards and transition probabilities between states. In situations where there is no previous reliable data, or reluctance to specify the prior distributions, the trial could employ an initial non-adaptive phase, followed by (CR)DP only after a sufficient amount of information has accumulated in the initial phase. This information could then be used to form the prior distribution for the subsequent adaptive (CR)DP phase.

Given the recent surge in papers on bandit-based RAR procedures (e.g. Ahuja and Birge, 2020; Chick et al., 2020; Kaibel and Biemann, 2021; Donahue and Sabo, 2021; Wang, 2021), this paper is a timely contribution to the literature, both from a methodological and practical perspective, where it is hoped that it will encourage others to provide a thorough consideration of practicalities when developing new methods. In upcoming work, currently under preparation, we further extend the (CR)DP model to incorporate information from patients whilst in the pipeline, instead of waiting until their responses have been observed.

## Supplementary Material

File 1

## Figures and Tables

**Table 1:** The success probability estimates, ${\hat{\theta }}_{A}$ and ${\hat{\theta }}_{B}$ for treatments *A* and *B*, respectively, compared to their true values, *θ_A_* and *θ_B_*, following CRDP and DRPWR with a fixed delay. These results correspond to the scenarios in which *n* = 75, *θ_A_* = 0.5 and *θ_B_* ∈ (0.1, 0.9) for a fixed delay of 5 (upper table) and 25 (lower table).

True		CRDP with delay 5				DRPWR with delay 5			
*θ_A_*	*θ_B_*	${\hat{\theta }}_{A}$	${\hat{\theta }}_{B}$	${\hat{\theta }}_{A}-{\hat{\theta }}_{B}$	Bias	${\hat{\theta }}_{A}$	${\hat{\theta }}_{B}$	${\hat{\theta }}_{A}-{\hat{\theta }}_{B}$	Bias
0.500	0.100	0.499853	0.096223	0.403630	0.003630	0.496576	0.097737	0.398840	−0.001160
0.500	0.200	0.497806	0.188783	0.309024	0.009024	0.496257	0.196250	0.300007	0.000007
0.500	0.300	0.491774	0.278339	0.213435	0.013435	0.495755	0.295181	0.200574	0.000574
0.500	0.400	0.480684	0.369758	0.110926	0.010926	0.495331	0.394947	0.100384	0.000384
0.500	0.500	0.470749	0.470066	0.000684	0.000684	0.494164	0.494433	−0.000269	−0.000269
0.500	0.600	0.469279	0.579858	−0.110578	−0.010578	0.492965	0.594547	−0.101582	−0.001582
0.500	0.700	0.477296	0.691518	−0.214222	−0.014222	0.490896	0.695328	−0.204432	−0.004432
0.500	0.800	0.487732	0.797777	−0.310045	−0.010045	0.487573	0.796879	−0.309306	−0.009306
0.500	0.900	0.495412	0.899759	−0.404347	−0.004347	0.480832	0.898330	−0.417498	−0.017498
True		CRDP with delay 25				DRPWR with delay 25			
*θ_A_*	*θ_B_*	${\hat{\theta }}_{A}$	${\hat{\theta }}_{B}$	${\hat{\theta }}_{A}-{\hat{\theta }}_{B}$	Bias	${\hat{\theta }}_{A}$	${\hat{\theta }}_{B}$	${\hat{\theta }}_{A}-{\hat{\theta }}_{B}$	Bias
0.500	0.100	0.499554	0.097617	0.401938	0.001938	0.497334	0.097964	0.399370	−0.000630
0.500	0.200	0.497649	0.193748	0.303900	0.003900	0.497083	0.196814	0.300270	0.000270
0.500	0.300	0.493642	0.288373	0.205269	0.005269	0.496828	0.296256	0.200572	0.000572
0.500	0.400	0.488466	0.384723	0.103742	0.003742	0.496502	0.396201	0.100300	0.000300
0.500	0.500	0.484371	0.484043	0.000329	0.000329	0.496184	0.496505	−0.000321	−0.000321
0.500	0.600	0.483978	0.588435	−0.104456	−0.004456	0.495625	0.596536	−0.100910	−0.000910
0.500	0.700	0.487662	0.693864	−0.206202	−0.006202	0.494818	0.697139	−0.202321	−0.002321
0.500	0.800	0.492379	0.797834	−0.305455	−0.005455	0.494015	0.798157	−0.304142	−0.004142
0.500	0.900	0.496040	0.899640	−0.403599	−0.003599	0.492782	0.898929	−0.406147	−0.006147
