# Supplementary material for: Generalisations of a Bayesian decision-theoretic randomisation procedure and the impact of delayed responses
Source: Comput Stat Data Anal. Author manuscript; Available in PMC 2022 Nov 1. (PMC7612844; doi:10.1016/j.csda.2021.107407)
Supplement: File 1 [file EMS144253-supplement-File_1.pdf]

in this area include Villar and Rosenberger (2018) and Villar et al. (2018). Alternatively, one may consider using block RAR to reduce the bias caused by patient drift (see e.g. Magirr, 2011).

Accrual (or selection) bias may also contribute to heterogeneity in patient recruitment over time. For example, with CRDP, patients may prefer to enter the trial earlier since, as we have seen in Section 4, patients entering the trial later may be more likely randomised to the inferior treatment in order to satisfy the constraint specified by CRDP. However, typically in response-adaptive trials, it is more desirable for patients to enter the trial later because, that way, their probability of being randomised to the better treatment will be higher. This highlights that, regardless of the constraint, this type of bias still poses a problem. The introduction of the constraint in CRDP may even circumvent, or at least mitigate, the effect of accrual bias since there is no longer an obvious "desirable" stage at which to enter the trial. It may even be the case that patients refuse to be allocated to a particular treatment or drop out, resulting in fewer patients on one arm. However, this is a concern in all studies and is typically mitigated through blinding and intention-to-treat analysis.

All the results presented in this paper assume a uniform prior for the unknown success probabilities of each arm. However, one could also consider an informative prior based on data from previous trials or expert opinion (Dallow et al., 2018; Williams et al., 2021), for example. The (CR)DP procedure also allows for implementing a decreasingly informative prior (see Donahue and Sabo, 2021) by modifying the rewards and transition probabilities between states. In situations where there is no previous reliable data, or reluctance to specify the prior distributions, the trial could employ an initial non-adaptive phase, followed by (CR)DP only after a sufficient amount of information has accumulated in the initial phase. This information could then be used to form the prior distribution for the subsequent adaptive (CR)DP phase.

Given the recent surge in papers on bandit-based RAR procedures (e.g. Ahuja and Birge, 2020; Chick et al., 2020; Kaibel and Biemann, 2021; Donahue and Sabo, 2021; Wang, 2021), this paper is a timely contribution to the literature, both from a methodological and practical perspective, where it is hoped that it will encourage others to provide a thorough consideration of practicalities when developing new methods. In upcoming work, currently under preparation, we further extend the (CR)DP model to incorporate information from patients whilst in the pipeline, instead of waiting until their responses have been observed.

Acknowledgements

This paper is based on parts of the Ph.D. thesis by Williamson (2020). The first author would like to thank Prof. James Wason for useful comments on this work and the support of the *Engineering and Physical Sciences Research Council* funding received through the STOR-i Centre for Doctoral Training, Lancaster University. (EP/H023151/1). Prof. Thomas Jaki received funding from the *Medical Research Council* (MC_UU_00002/14).

Appendix A

A.1. Backward recursion algorithm for generalised CRDP

The backward recursion algorithm from the theory of dynamic programming runs backward in time step variable t, starting at the end of the time horizon, i.e. $t = T$, and decreasing to $t = 0$. At each time step t, it runs through all of the joint states $\boldsymbol{z} = (s_A, f_A, s_B, f_B)$ reachable at that time step, i.e. those satisfying $s_A + f_A + s_B + f_B = t$. Note that at time step t, we assume that t patients have been allocated using this procedure and t outcomes have been observed. However, in the case of delayed responses with fixed delay d, for example, we would only have observed $t - d$ outcomes from patients allocated using this procedure, and another d outcomes from patients allocated during the initial phase with equal fixed randomisation.

If $t = T$, there is no reward to receive from allocating patients because no more patients will arrive. Thus, we only consider the penalties and, consequently, $\mathcal{F}_T(\boldsymbol{z}) = Q(\boldsymbol{z})$ for all $\boldsymbol{z}$ that sum to T.

For $t = T - 1$ to $t = 0$, the value-to-go functions $\mathcal{F}_t$ satisfy the Bellman equation which allows them to be expressed recursively as functions of $\mathcal{F}_{t+1}$'s. Suppose now that $\boldsymbol{z}$ is such that $s_A + f_A + s_B + f_B = t$. Denote the unit vector of length four by $\boldsymbol{e}_i$, with the i-th element equal to 1. We decompose the time step into three sub-steps: (1) pre-decision, i.e. before making the action choice when penalty-involving reward $Q(\boldsymbol{z})$ is incurred, (2) post-decision, i.e. after making the action choice, but before effectuating the randomisation, and (3) post-allocation, i.e. after effectuating the randomisation resulting in a patient allocation, during which a patient response is observed and the trial state is updated at the beginning of the next time step.

Proceeding backwards, we first define the post-allocation quantities of the value-to-go function. If treatment A is allocated to the next patient, then the value-to-go function under an optimal policy is

$$\mathcal{F}_t^A(\boldsymbol{z}) = \frac{\widetilde{s}_A}{\widetilde{s}_A + \widetilde{f}_A} \cdot (1 + \mathcal{F}_{t+1}(\boldsymbol{z} + \boldsymbol{e}_1)) + \frac{\widetilde{f}_A}{\widetilde{s}_A + \widetilde{f}_A} \cdot \mathcal{F}_{t+1}(\boldsymbol{z} + \boldsymbol{e}_2),$$

where $\widetilde{s}_j = s_{j,0} + s_j$ and $\widetilde{f}_j = f_{j,0} + f_j$ for treatment j represents the prior information and observed data combined.

Alternatively, if treatment B is allocated to the next patient, then the value-to-go function under an optimal policy is

$$\mathcal{F}_t^B(\boldsymbol{z}) = \frac{\widetilde{s}_B}{\widetilde{s}_B + \widetilde{f}_B} \cdot (1 + \mathcal{F}_{t+1}(\boldsymbol{z} + \boldsymbol{e}_3)) + \frac{\widetilde{f}_B}{\widetilde{s}_B + \widetilde{f}_B} \cdot \mathcal{F}_{t+1}(\boldsymbol{z} + \boldsymbol{e}_4).$$

Second, we define the post-decision quantities of the value-to-go function. If action $a = 1$, then the value-to-go function under an optimal policy is $\mathcal{F}_t^1(\boldsymbol{z}) = p_{A,t} \cdot \mathcal{F}_t^A(\boldsymbol{z}) + (1 - p_{A,t}) \cdot \mathcal{F}_t^B(\boldsymbol{z})$, and analogously when action $a = 2$, that is, $\mathcal{F}_t^2(\boldsymbol{z}) = (1 - p_{B,t}) \cdot \mathcal{F}_t^A(\boldsymbol{z}) + p_{B,t} \cdot \mathcal{F}_t^B(\boldsymbol{z})$.

Finally, the pre-decision quantities of the value-to-go function are defined as

$$\mathcal{F}_t(\boldsymbol{z}) = Q(\boldsymbol{z}) + \max\left\{\mathcal{F}_t^1(\boldsymbol{z}),\ \mathcal{F}_t^2(\boldsymbol{z})\right\}.$$

Thus, if $\mathcal{F}_t^1(\boldsymbol{z}) > \mathcal{F}_t^2(\boldsymbol{z})$, then it is optimal to employ action 1, and vice versa. If they are equal, then both actions are optimal choices, and one would equally randomise between them to avoid any systematic allocation bias.

A.2. Simulation results for DP with delayed responses

Fig. A.8 presents performance measures for DP with fixed delay and Fig. A.9 presents performance measures for DP with random delay.

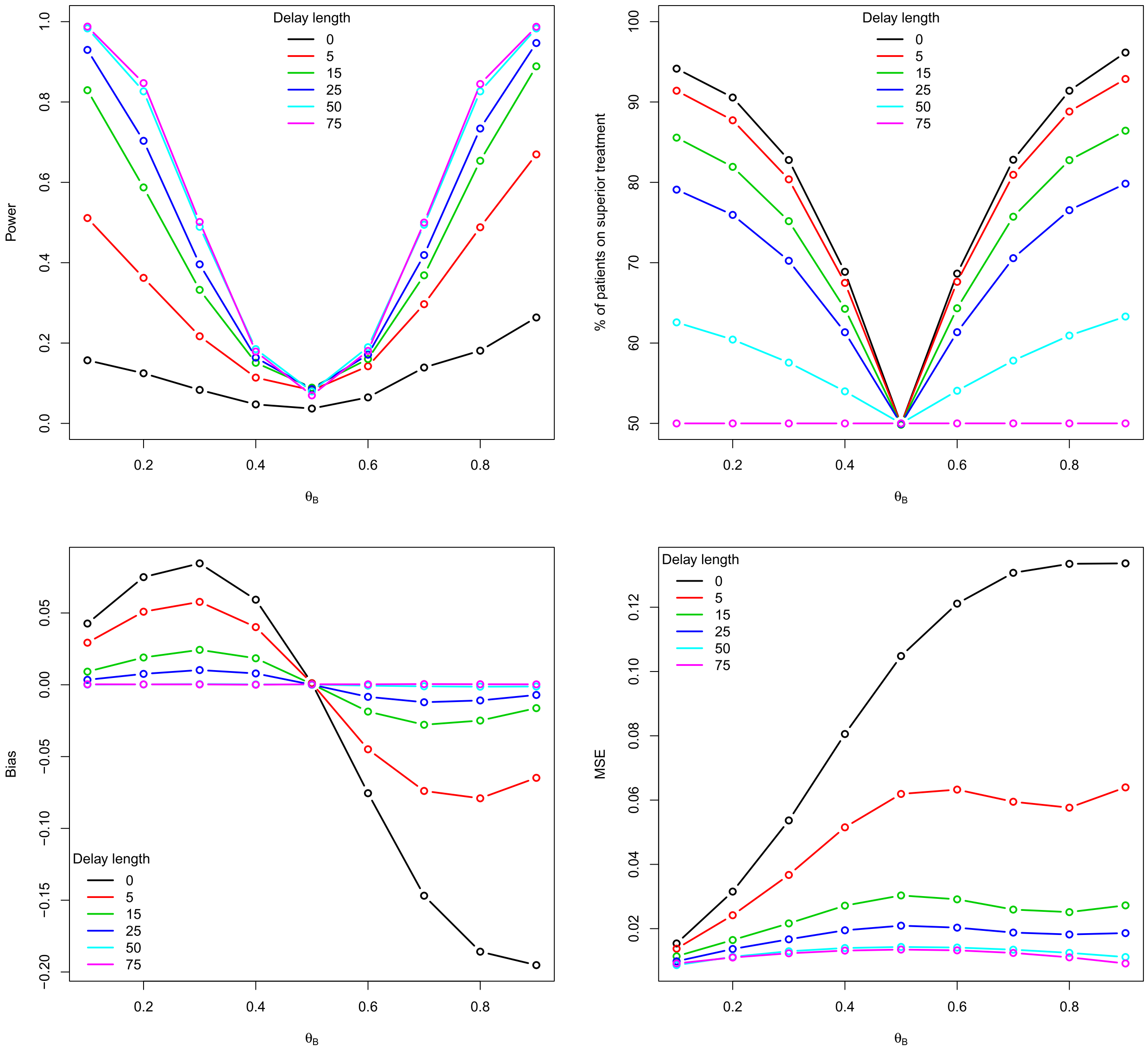

Fig. A.8. Power/type I error, % of patients on the superior treatment, the average bias and MSE of the treatment effect estimator for DP when $n = 75$, $\theta_A = 0.5$ and $\theta_B \in (0.1, 0.9)$ for different fixed delay lengths (estimated over 100,000 simulations).

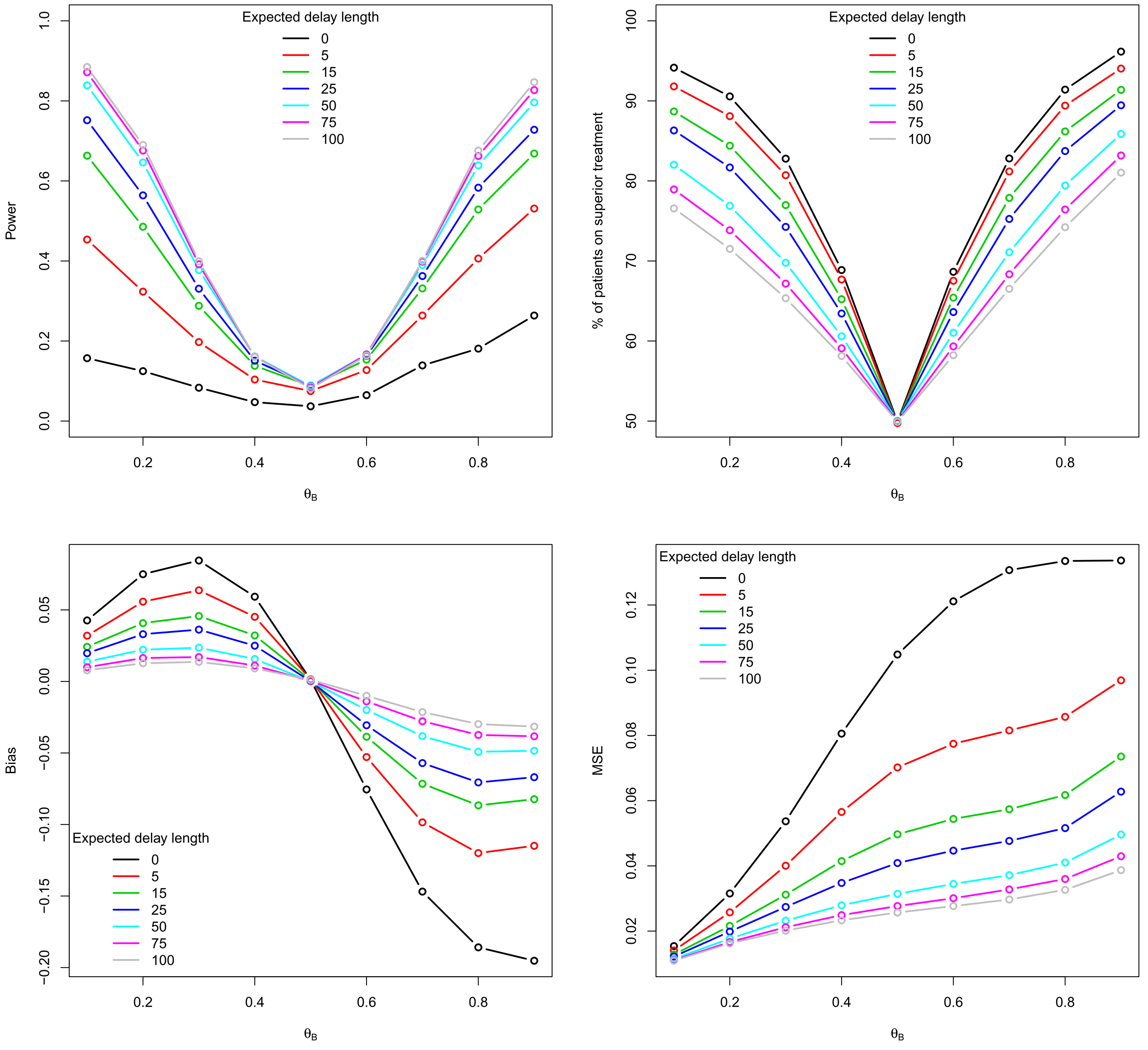

Fig. A.9. Power/type I error, % of patients on the superior treatment, the average bias and MSE of the treatment effect estimator for DP when $n = 75$, $\theta_A = 0.5$ and $\theta_B \in (0.1, 0.9)$ for different expected delay lengths (estimated over 100,000 simulations).

Similar patterns of results are observed for the DP procedure as for the CRDP procedure, but an increased delay brings much higher benefits for statistical operating characteristics in the DP case. This is because the baseline statistical performance of DP is very poor due to the lack of randomisation and constraining, meaning a greater level of imbalance can occur (note that the scale of the bias and MSE plots for the DP is much larger than that used for the corresponding CRDP plots).

Consider the fixed delay case in Fig. A.8 with $\theta_B = 0.1$. While the no delay case has a power around 0.17, a delay of $d = 5$ increases it to around 0.51 and a delay of $d = 15$ to 0.83. At the same time, the percentage of patients on the superior treatment decreases from 94% to 91% and 86%, respectively. A delay of around $d = 22$ introduces sufficient balancing effects (on average, at least 11 observations on each arm) to bring DP to perform akin to CRDP in the no delay case (in which the degree of constraining penalises end-of-trial states with ≤ 11 observations). When the delay is 25 (one third of the trial size), there is a loss of approximately 15% in patient benefit relative to the value attained in the no delay case. However, the percentage of patients on the superior treatment is still approximately 30% larger than with equal fixed randomisation. In terms of the power, a delay of 25 increases it to around 0.93 (almost 80% greater than when there is no delay), which is very close to the power obtained by equal fixed randomisation. Therefore, by introducing a delay in response, although the DP procedure is now adapting based on reduced information, it continues to allocate a considerably

large percentage of patients to the superior treatment whilst achieving a substantially improved power and bias/MSE over the no delay case.

A.3. Simulation results for DRPWR

Fig. A.10 and Fig. A.11 present performance measures for DRPWR with fixed delay and random delay, respectively. Note that the performance of DRPWR is not symmetric about $\theta_B = 0.5$ due to the increased chance of observing a positive response as the success probability increases.

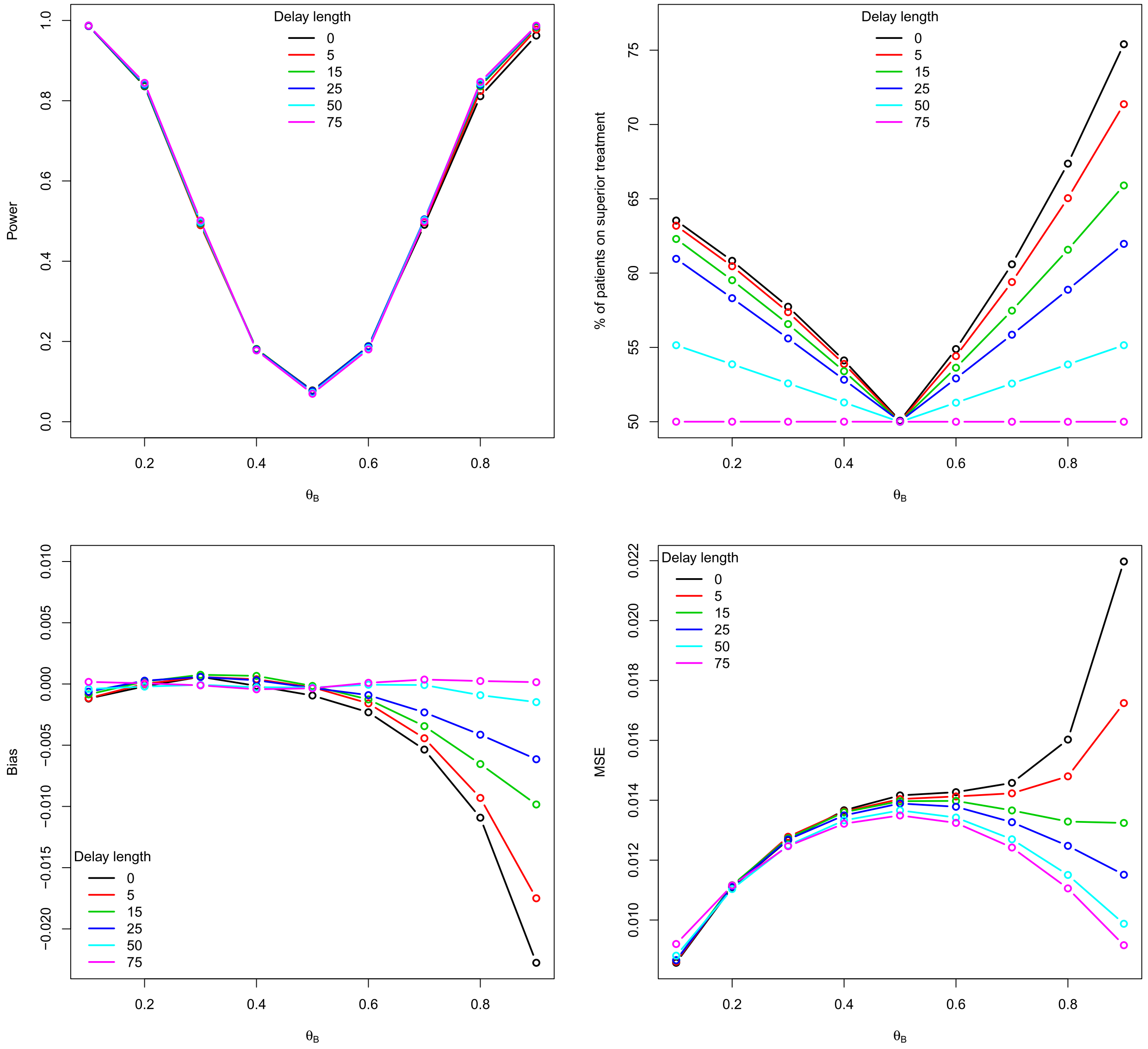

Fig. A.10. The changes in power (and type I error), % of patients on the superior treatment, the average bias and MSE of the treatment effect estimator for the DRPWR when $n = 75$, $\theta_A = 0.5$ and $\theta_B \in (0.1, 0.9)$ for different fixed delay lengths (estimated over 100,000 simulations).

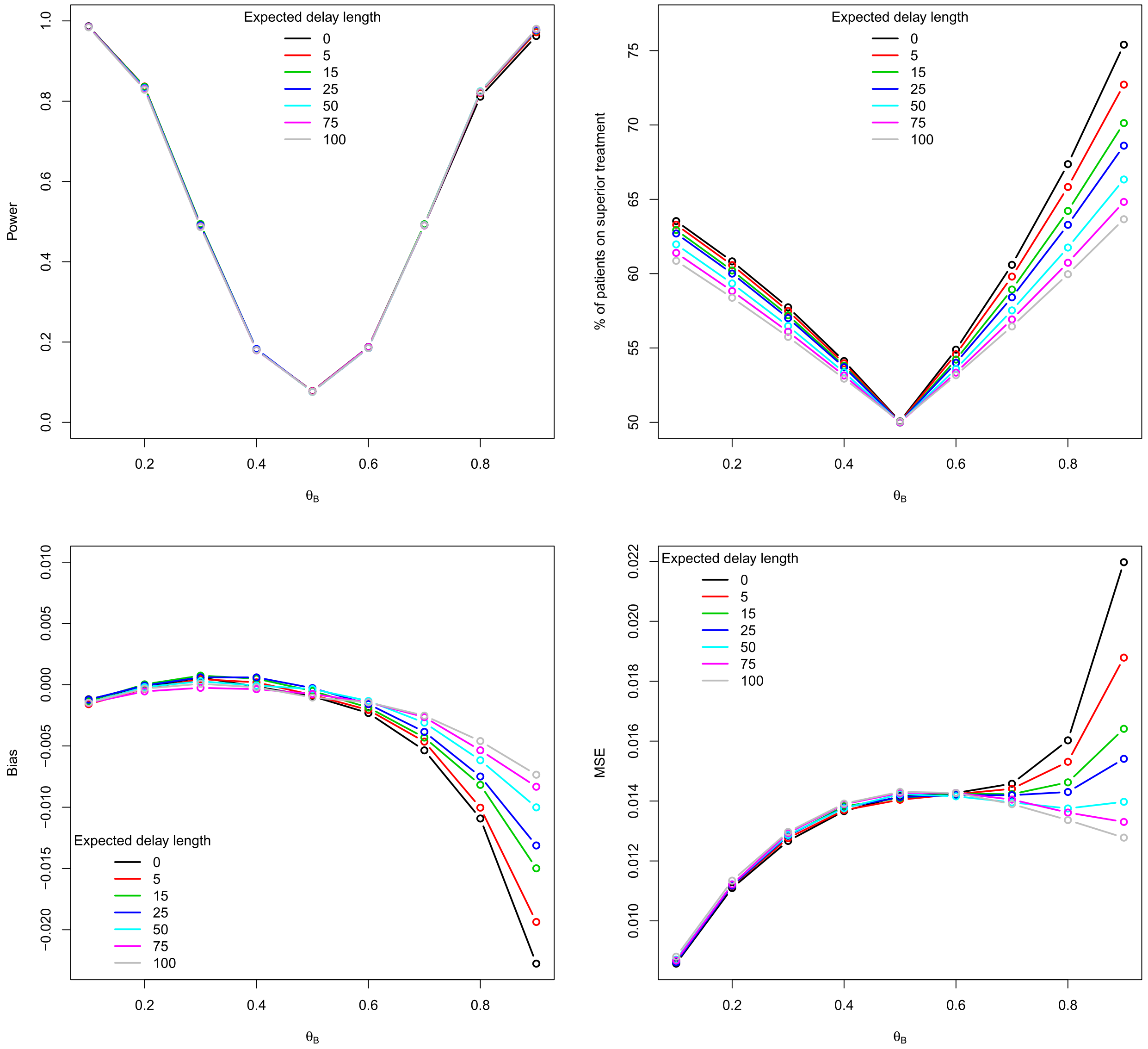

Fig. A.11. Power/type I error, % of patients on the superior treatment, the average bias and MSE of the treatment effect estimator for the DRPWR when $n = 75$, $\theta_A = 0.5$ and $\theta_B \in (0.1, 0.9)$ for different expected delay lengths (estimated over 100,000 simulations).

A.4. Comparison of (CR)DP to DRPWR

Fig. A.12 compares the performance of (CR)DP to the DRPWR in trials with a *fixed* delay and no treatment difference.

The first plot in Fig. A.12 illustrates the changes in type I error rates for the (CR)DP and DRPWR as the delay increases. The type I error rate of (CR)DP appears to first increase and then decrease with d because there are two opposing forces involved: conservatism of the Fisher's exact test (especially for small sample sizes) and increased error caused by the RAR. Recall that the desired significance level is 0.1. However, under equal randomisation, Fisher's exact test is not reaching that level due to the conservatism of the test and the attained level is in fact 0.07 (represented by the red dashed line). As the delay length increases, (CR)DP behaves similarly to equal randomisation and, thus, the type I error rate approaches the attained significance level of 0.07 (which is why we observe a decrease). If the test was attaining the nominal level of 0.1, then we would observe inflation of the type I error due to the RAR.

The type I error rates for the DRPWR are consistently smaller, albeit very slightly, than those for (CR)DP (with delay) until around $d = 60$, after which they perform similarly. Since the treatments have the same success rates, the percentage of patients allocated to either treatment behaves accordingly (close to 50%) and the bias values lie within $(-0.001, 0.001)$ irrespective of the procedure or delay length.

Fig. A.13 compares the performance of (CR)DP to the DRPWR in trials with a *random* delay and no treatment difference.

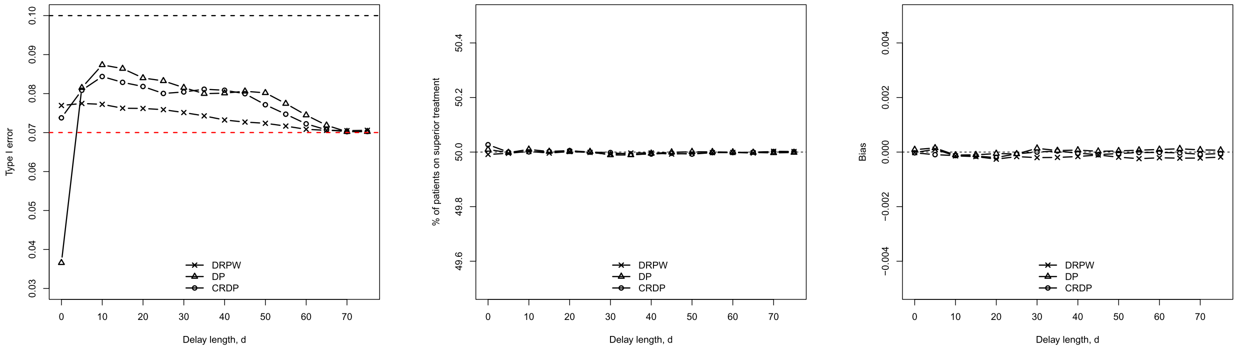

Fig. A.12. The changes in type I error, % of patients on the superior treatment and the average bias of the treatment effect estimator for (CR)DP and DRPWR as the length of the fixed delay increases, when $n = 75$, $\theta_A = \theta_B = 0.5$ (estimated over 100,000 simulations). NB: The black and red horizontal dashed lines represent the nominal (0.1) and attained (0.07) significance levels, respectively.

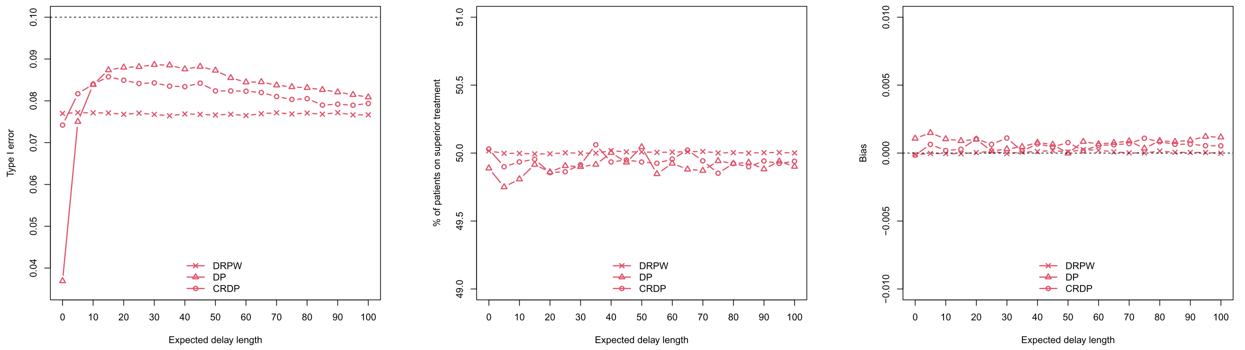

Fig. A.13. The changes in type I error, % of patients on the superior treatment and the average bias of the treatment effect estimator for (CR)DP and DRPWR as the expected delay length increases, when $n = 75$ and $\theta_A = \theta_B = 0.5$ (estimated over 100,000 simulations).

The first plot illustrates the changes in type I error rates for (CR)DP and DRPWR as the expected delay increases. As in the fixed delay case, after an initial increase for (CR)DP, the type I error rate then decreases gradually. In contrast, the type I error for DRPWR remains relatively constant.

A.5. Comparison of fixed and random delays on (CR)DP

Here, we compare the performance measures of the (CR)DP with a fixed delay versus (CR)DP with a random delay for a specific scenario in which $\theta_A = 0.5$ and $\theta_B = 0.1$. We have calibrated the random delays so that we expect them to be the same length, on average, as the fixed delays. We use this comparison purely for illustrative purposes to highlight the differences that can occur as a result of the delay being random rather than fixed. Fig. A.14 shows that there is a smaller power, more patients on the superior treatment and a larger bias observed.

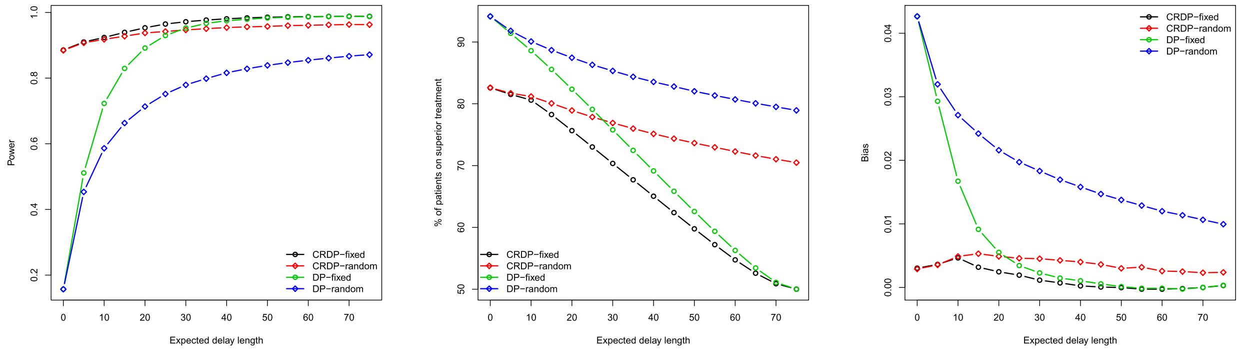

Fig. A.14. The changes in power, % of patients on the superior treatment and the average bias of the treatment effect estimator for CRDP as the fixed/expected delay length increases, when $n = 75$, $\theta_A = 0.5$ and $\theta_B = 0.1$ (estimated over 100,000 simulations).

It is interesting to note that for $d = n$, the percentage of patients on the superior treatment is 50% when the delay is fixed, as expected, but closer to 70% for CRDP and 79% for DP if it is random (see the middle plot in Fig. A.14). This is because there will be some patients with a small (or no) delay, by random chance, in which case the (CR)DP procedure still adapts relatively quickly and leads to a higher patient benefit (see Fig. A.15). Similarly for the bias, which is not converging to 0 as quickly as in the fixed delay case.

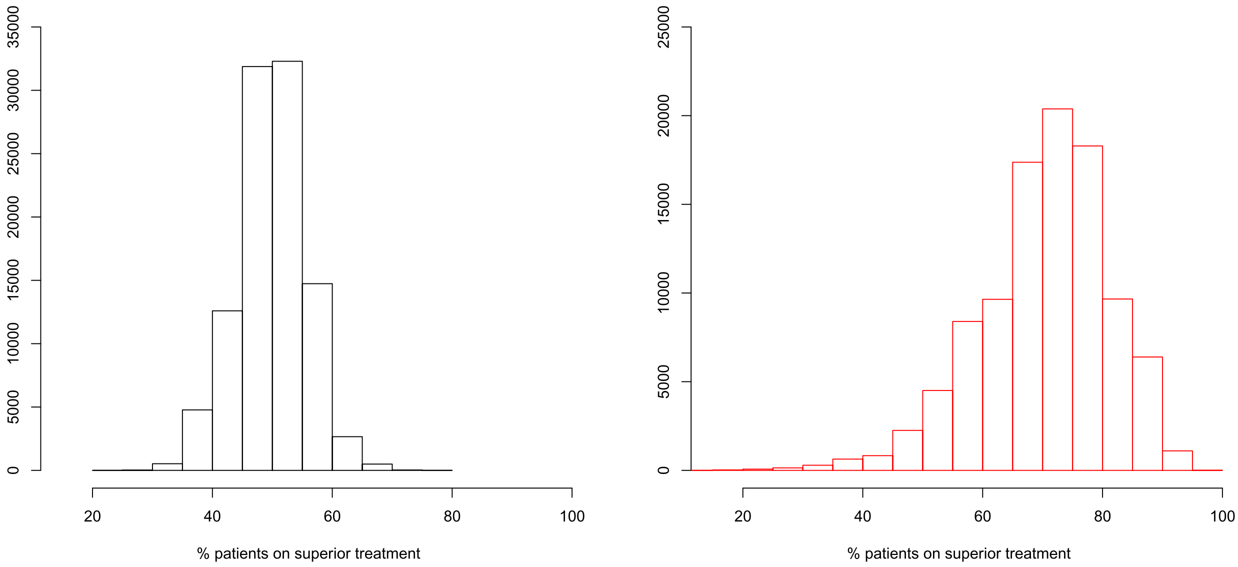

(a) CRDP with fixed delay (b) CRDP with random delay

Fig. A.15. Histograms showing the distribution of 100,000 simulations for the % of patients on the superior treatment when the fixed/expected delay length is 75, $n = 75$, $\theta_A = 0.5$ and $\theta_B = 0.1$.

Moreover, when the (expected) delay is small ($d = 0$ and $d = 5$), we observe that the performance of CRDP is similar regardless of whether the delay is fixed or random. However, for larger (expected) delays, random delays affect the performance similarly to much shorter fixed delays, e.g. random $d = 25$ is akin to fixed $d = 15$, random $d = 50$ is akin to fixed $d = 25$, and random $d = 100$ seems akin to fixed $d = 35$.

A.6. Adjusting the time horizon of DP

Fig. A.16 presents performance measures for DP (with time horizon $T = n$) and DP-TH (with time horizon $T = n - d$). Fig. A.17 illustrates the effect of changing the delay length d on the average allocation probabilities when using DP with different time horizons.

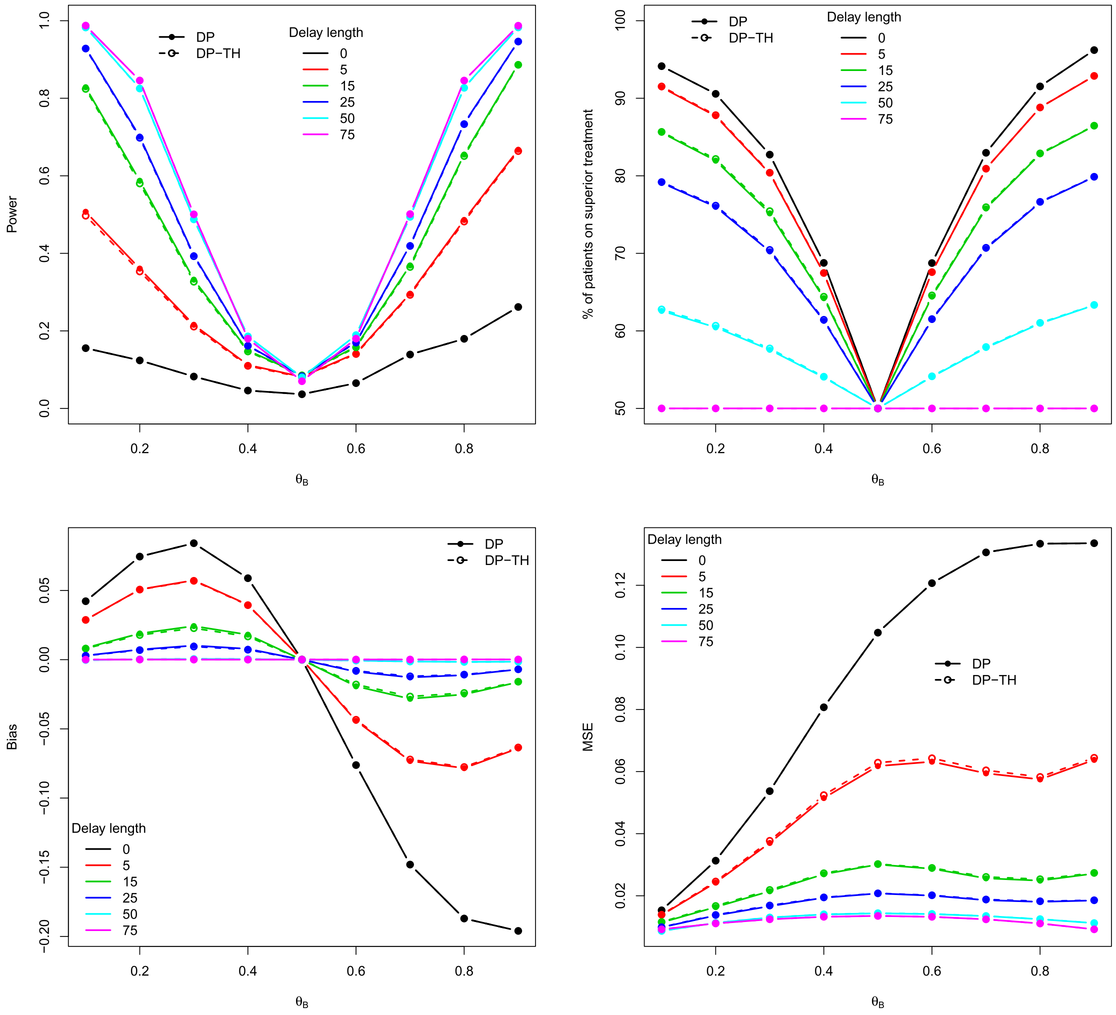

Fig. A.16. Power/type I error, % of patients on the superior treatment, the average bias and MSE of the treatment effect estimator for DP and DP-TH when $n = 75$, $\theta_A = 0.5$ and $\theta_B \in (0.1, 0.9)$ for different delay lengths d (estimated over 1, 000, 000 simulations).

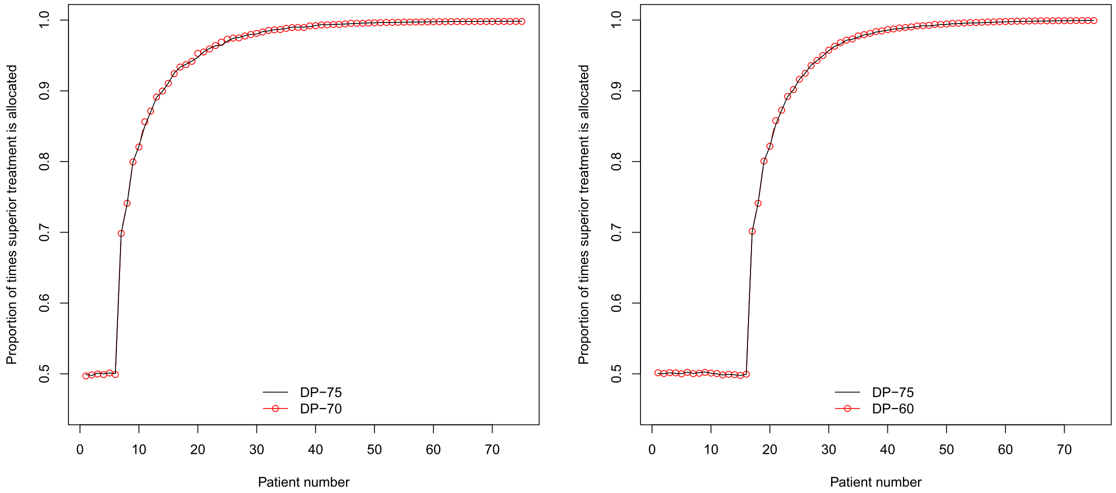

(a) $d = 5$ (b) $d = 15$

Fig. A.17. Probability of allocating a patient to the superior treatment when $\theta_A = 0.5$ and $\theta_B = 0.9$ in a trial of size $n = 75$ (estimated over 1, 000, 000 simulations). The black and red lines correspond to DP with time horizons $T = n$ and $T = n - d$, respectively.
